# Complexes of Bifunctional DO3A-N-(α-amino)propinate Ligands with Mg(II), Ca(II), Cu(II), Zn(II), and Lanthanide(III) Ions: Thermodynamic Stability, Formation and Dissociation Kinetics, and Solution Dynamic NMR Studies

**DOI:** 10.3390/molecules26164956

**Published:** 2021-08-16

**Authors:** Zoltán Garda, Tamara Kócs, István Bányai, José A. Martins, Ferenc Krisztián Kálmán, Imre Tóth, Carlos F. G. C. Geraldes, Gyula Tircsó

**Affiliations:** 1 Department of Physical Chemistry, Faculty of Science and Technology, University of Debrecen, H-4010 Debrecen, Hungary; garda.zoltan@science.unideb.hu (Z.G.); tamra86@gmail.com (T.K.); banyai.istvan@science.unideb.hu (I.B.); kalman.ferenc@science.unideb.hu (F.K.K.); imre.toth@science.unideb.hu (I.T.); 2 Centro de Química, Campus de Gualtar, Universidade do Minho, 4710-057 Braga, Portugal; jmartins@quimica.uminho.pt; 3 Department of Life Sciences and Coimbra Chemistry Centre, Faculty of Science and Technology, University of Coimbra, 3000-393 Coimbra, Portugal; 4 CIBIT/ICNAS-Instituto de Ciências Nucleares Aplicadas à Saúde, Pólo das Ciências da Saúde, Azinhaga de Santa Comba, 3000-548 Coimbra, Portugal

**Keywords:** bifunctional ligands (BFCs), complexes, equilibrium, formation and dissociation kinetics, dynamic NMR

## Abstract

The thermodynamic, kinetic, and structural properties of Ln^3+^ complexes with the bifunctional DO3A-ACE^4−^ ligand and its amide derivative DO3A-BACE^4−^ (modelling the case where DO3A-ACE^4−^ ligand binds to vector molecules) have been studied in order to confirm the usefulness of the corresponding Gd^3+^ complexes as relaxation labels of targeted MRI contrast agents. The stability constants of the Mg^2+^ and Ca^2+^ complexes of DO3A-ACE^4−^ and DO3A-BACE^4−^ complexes are lower than for DOTA^4−^ and DO3A^3−^, while the Zn^2+^ and Cu^2+^ complexes have similar and higher stability than for DOTA^4−^ and DO3A^3−^ complexes. The stability constants of the Ln(DO3A-BACE)^−^ complexes increase from Ce^3+^ to Gd^3+^ but remain practically constant for the late Ln^3+^ ions (represented by Yb^3+^). The stability constants of the Ln(DO3A-ACE)^4−^ and Ln(DO3A-BACE)^4−^ complexes are several orders of magnitude lower than those of the corresponding DOTA^4−^ and DO3A^3−^ complexes. The formation rate of Eu(DO3A-ACE)^−^ is one order of magnitude slower than for Eu(DOTA)^−^, due to the presence of the protonated amine group, which destabilizes the protonated intermediate complex. This protonated group causes the Ln(DO3A-ACE)^−^ complexes to dissociate several orders of magnitude faster than Ln(DOTA)^−^ and its absence in the Ln(DO3A-BACE)^−^ complexes results in inertness similar to Ln(DOTA)^−^ (as judged by the rate constants of acid assisted dissociation). The ^1^H NMR spectra of the diamagnetic Y(DO3A-ACE)^−^ and Y(DO3A-BACE)^−^ reflect the slow dynamics at low temperatures of the intramolecular isomerization process between the SA pair of enantiomers, R-*Λ*(*λλλλ*) and S-*Δ*(*δδδδ*). The conformation of the C_α_-substituted pendant arm is different in the two complexes, where the bulky substituent is further away from the macrocyclic ring in Y(DO3A-BACE)^−^ than the amino group in Y(DO3A-ACE)^−^ to minimize steric hindrance. The temperature dependence of the spectra reflects slower ring motions than pendant arms rearrangements in both complexes. Although losing some thermodynamic stability relative to Gd(DOTA)^−^, Gd(DO3A-BACE)^−^ is still quite inert, indicating the usefulness of the bifunctional DO3A-ACE^4−^ in the design of GBCAs and Ln^3+^-based tags for protein structural NMR analysis.

## 1. Introduction

The ultimate goal of modern diagnostic and molecular imaging techniques is the visualization of biological processes that occur at the cellular and molecular levels. Magnetic resonance imaging (MRI) is one of the most powerful clinical imaging tools available due to its superb spatial resolution and the capability of generating excellent soft tissue contrast using different mechanisms, the most common of which results from intrinsic differences in the relaxation times (*T*_1,2_) of tissue water protons. The contrast between normal and diseased tissues can be dramatically improved by the use of contrast agents (CAs), namely paramagnetic Gd^3+^-based complexes or superparamagnetic iron-oxide based nanoparticles (*T*_1_- and *T*_2_-shortening agents, respectively [1,2,3]. The Gd^3+^-based contrast agents (GBCAs) currently used in the clinics for *T*_1_-weighed imaging are anionic or neutral Gd^3+^ chelates of linear (DTPA-type) or macrocyclic (DOTA-type) poly(aminocarboxylate) ligands. 

To ensure their safety, the GBCA complexes are endowed with high thermodynamic stability and kinetic inertness to prevent in vivo release of the highly toxic free metal ion. However, the recently recognized rare but severe medical condition known as nephrogenic systemic fibrosis (NSF) has been associated with the administration of GBCAs to patients with significant renal disease [4,5], leading to in vivo Gd^3+^ release which has been detected mostly as GdPO_4_ deposits by means of elemental bioimaging and speciation analysis [6]. Even more recently, residual gadolinium has been detected in brain structures of patients with normal renal function after repeated application of GBCAs [7,8,9]. Most NSF cases have been associated with the use of linear GBCAs, particularly the neutral Gd(DTPA-bis(amides)) [8]. Lower thermodynamic stability and increased kinetic lability, coupled with slow kidney clearance, result in extensive complex demetallation in vivo [10]. Macrocyclic GBCAs are considered safe due to their higher thermodynamic stability and kinetic inertness [11].

Ways to generate effective contrast at lower doses include efficacy optimization and targeting of the GBCAs to sites of interest. Relaxivity (*r*_1,2_), which is the paramagnetic enhancement of water proton relaxation rates *R*_1,2_ (*R*_1,2_ = 1/*T*_1,2_) normalized to 1 mM concentration, is a measure of CA efficacy. The simultaneous optimization of the molecular parameters governing relaxivity, namely the rotational correlation time (*τ*_R_), the water exchange rate (*k*_ex_ = 1/*τ*_M_), and the electronic relaxation parameters of the complexes should lead to very high relaxivities [2].The strategies for tuning *τ*_R_ and *k*_ex_ to an optimal range of values to obtain high relaxivities at intermediate fields relevant for clinical MRI have found some success [12,13]. Slower tumbling rates (longer *τ*_R_) enhance the relaxivity at intermediate fields, which can be attained in different ways involving the increase of the molecular weight of the CA, its self-association or association with macromolecular or nanostructures [12,13]. Accelerating water exchange of GBCAs towards optimal *k*_ex_ values has been attained by enforcing steric compression around the water binding site on Gd^3+^ complexes with dissociatively activated exchange processes. This has been achieved by introduction of an extra methylene group between the amines of the ligands DOTA^4−^ and DTPA^5−^, leading to chelators TRITA^4−^ and EPTPA^5−^, respectively, whose Gd^3+^ chelates have *k*_ex_ values around two orders of magnitude higher than the parent Gd(DOTA)^−^ and Gd(DTPA)^2−^ [14,15,16]. Gd^3+^ complexes of ligands bearing a pendant propionate arm (DO3A-*N*prop^4−^, DTTA-*N*prop^4−^) have a moderate (one order of magnitude) water exchange acceleration in relation to Gd(DOTA)^−^ and Gd(DTPA)^2−^, in the ideal range for attaining high relaxivities at medium (60–100 MHz) frequencies [17]. However, these structural changes of the DOTA^4−^ and DTPA^5−^ framework lead in some extent to loss of thermodynamic stability and kinetic inertness of the corresponding Gd^3+^ chelates [18,19].

**Scheme 1:**
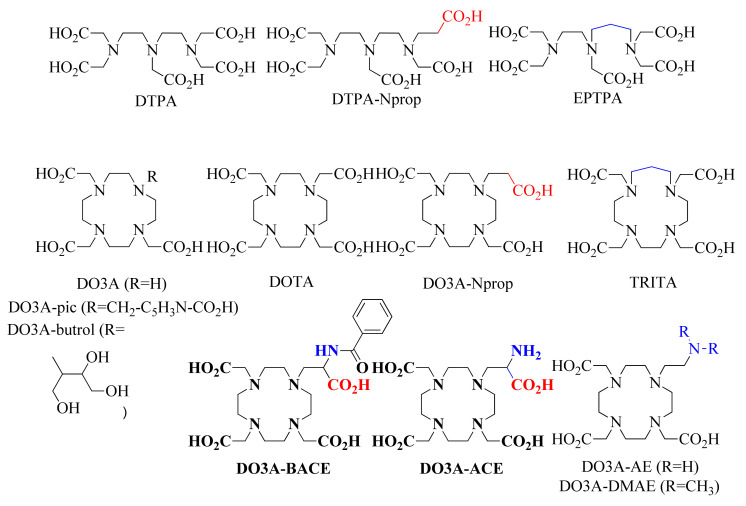
Chemical structures of H_4_DO3A-ACE^4−^, H_4_DO3A-BACE^4−^, and related DTPA and DOTA-type ligands discussed in the text.

Obtaining optimal targeted GBCAs involves designing bifunctional chelators endowed with conjugability and leading to Gd^3+^ chelates with high relaxivity, while maintaining high thermodynamic stability and kinetic inertness. We have recently reported the synthesis and in vitro and in vivo evaluation of Gd^3+^ complexes of the DO3A-N-(α-aminopropionate) chelator [20] and of some of its amide conjugates [21,22,23,24], (Scheme 1) as GBCAs for MRI, which have inspired other research groups [25]. The introduction of an amine group in the propionate pendant arm of DO3A-*N*prop and its conjugation with various targeting model molecules did not affect the optimized *k*_ex_ value, leading to high *r*_1_ relaxivities at intermediate fields upon slowing down their tumbling rates via self-association or binding to gold nanoparticles, as well as efficient positive contrast in *T*_1_-weighted images of small animals [21,22,23,24]. Thus, in this work, we describe a detailed evaluation of the thermodynamic stability of the complexes of DO3A-*N*-(α-aminopropionate) (DO3A-ACE^4−^, H_4_DO3A-ACE = 1-(2-amino-carboxyethyl)-4,7,10-tris-(caboxymethyl)-1,4,7,10-tetraazacyclododecane) and of its benzoylamide conjugate (DO3A-BACE^4−^, H_4_DO3A-BACE = 1-(2-benzylamido-carboxyethyl)-4,7,10-tris-(caboxymethyl)-1,4,7,10-tetraazacyclodode cane) (Scheme 1) with essential metal ions (Mg^2+^, Ca^2+^, Cu^2+^, and Zn^2+^) and the lanthanide ions Ce^3+^, Gd^3+^, and Yb^3+^, as well as the formation kinetics of the Eu^3+^ complexes and the dissociation kinetics of the Ce^3+^ and Gd^3+^ complexes, using pH-potentiometry, ^1^H relaxometry, and UV-Vis spectrophotometry. The solution structures of the corresponding Y^3+^ complexes were also studied by ^1^H NMR. The results were compared with those published for DOTA^4−^, DO3A^3−^, DO3A-*N*prop^4−^ DO3A-AE^3−^, and TRITA^4−^.

## 2. Results

### 2.1. Ligand Protonation Constants and Stability Constants of the Metal Complexes 

The protonation constants of DO3A-ACE^4−^ and DO3A-BACE^4−^ were obtained by pH-potentiometric titrations performed in the pH range 1.80–11.85 at an ionic strength of 0.15 M NaCl and 25 °C (Figures S1 and S2). The protonation constants *K*_i_^H^, defined in Equation (1) and calculated from the titration data, are given in Table 1, where they are compared with values published for the other tetraazacyclododecane carboxylate ligands DOTA^4−^, DO3A^3−^, DO3A-*N*-prop^4−^, and that of DO3A-AE^3−^.

In the case of DO3A-BACE^4−^, the protonation sequence of its basic sites is the same as for DOTA^4−^, DO3A^3−^, and DO3A-N-prop^4−^, namely the first two protonation steps occurring at two opposite amine nitrogen atoms of the macrocycle, followed by protonation of the carboxylate oxygens. The first two protonation constants are significantly lower than the corresponding values for the above ligands, possibly due to the weakening of the H-bond between a protonated ring nitrogen and the carboxylate group of its propionate side chain (which is responsible for the increased basicity of that nitrogen in those ligands) due to the steric hindrance of the bulky substituent at the neighboring amino group. In DO3A-ACE^4−^, there are three protonation constants, rather than two, in the 10.0–8.5 range, due to the presence of a primary amine at the propionate side chain. This range of values is compatible with the log *K*^H^ of the amino group of alanine (9.87), as well as the log *K*^H^ of the DO3A-AE^3−^ free amino group (8.80) [32]. However, the protonation sequence of the first three basic sites cannot be established without the data from a proton NMR pH titration. The other two protonation constants are again significantly lower than the values for the DOTA-type ligands and DO3A-AE^3−^ [32], indicating that the H-bond established between the protonated ring nitrogen and either the carboxylate group (as in DO3A-N-prop^4−^) [19] or the amino group (as in DO3A-AE^3−^) [32] is weakened by the steric hindrance of the other substituent. The last two protonation constants of DO3A-ACE^4−^ are in agreement with the last three of DO3A-BACE^4−^, and correspond to protonation of unsubstituted acetate oxygens. The large decrease of the first protonation constant of the ring nitrogens in the two ligands may also partly be attributed to the non-corrected effect of Na^+^ present in the medium at 0.15 M concentration, i.e., the NaL complexes are in the fully deprotonated form, as observed before in the case of DOTA^4−^ [29,31]. The decreased overall basicity of the two aminated ligands is reflected in the much lower values of the sum of the first four protonation constants (Σ log *K*_4_^H^) than for the other ligands shown in Table 1.

The stability constants, log *K*_ML_, and the protonation constants, log *K*_MHiL_, of the complexes with several alkali earth, transition, and lanthanide metal ions (Equations (2) and (3)) are also determined by potentiometric titrations (Figures S1 and S2). They are listed and compared with the corresponding values for DOTA^4−^, DO3A^3−^, TRITA^4−^, and DO3A-Nprop^4−^ in Table 2 and Table 3.

**Figure 1:**
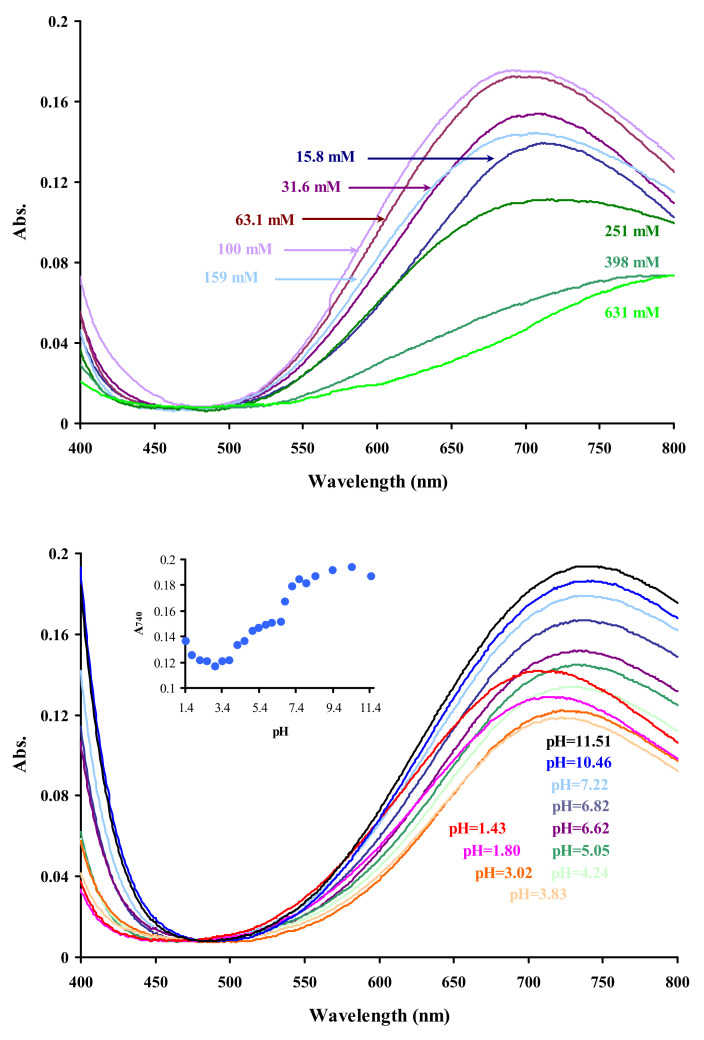
Absorption spectra of the Cu^2+^–DO3A-ACE^4−^–H^+^ system ([HCl] + [NaCl] = 1.0 M; [Cu^2+^] = [DO3A-ACE^4−^] = 1.94 mM, l = 1 cm, 25 °C). The top figure shows the batch samples prepared in the acid concentration range of 15.8 to 631 mM, while the other one shows the spectra obtained in the pH range of pH = 1.43 to 11.51. The insert indicates the absorbance measured at 740 nm as a function of pH.

For the alkali earth and transition metal ions studied, the trend of decreasing complex stability is Cu^2+^ > Zn^2+^ > Ca^2+^ > Mg^2+^ for DO3A-ACE^4−^ and DO3A-BACE^4−^, which remains the same as observed before for the complexes of DOTA^4−^, DO3A^3−^, DO3A-Nprop^4−^, and DO3A-AE^3−^ (Table 2) [19,27,33,34,35,36,37]. While the Cu^2+^ and Mg^2+^ complexes of DO3A-BACE^4−^ are more stable than those of DO3A-ACE^4−^, the opposite trend of stability constants was observed for Zn^2+^ and Ca^2+^, highlighting the inverse effect of a bulky neutral benzylamide substituent in the first ligand and the positively charged amino group in the second ligand on the relative stabilities of the two pairs of complexes. The Cu^2+^ coordination geometry of Cu(DOTA)^2−^ in its X-ray crystal structure is distorted octahedral, with coordination of the four macrocyclic nitrogens and two carboxylate oxygens in trans position, with the other two carboxylates remaining uncoordinated [37]. It is interesting to notice that the Cu^2+^ complexes of the DO3A-ACE^4−^ and DO3A-BACE^4−^ ligands are seemingly one to two orders of magnitude more stable than the Cu(DOTA)^2−^. This effect is opposite to the strong destabilization effect of the propionate pendant arm in the Cu(DO3A-Nprop)^4−^ complex [19]. However, it was found for numerous DOTA derivatives that the stability constants determined solely by pH-potentiometic titration, which appeared to be the standard procedure for determining stability constants of Cu^2+^ complexes, are underestimated, as the majority of the multiply protonated forms of these complexes are formed at 100% extent already near pH = 1.75, which is considered to be the lower limit for the pH-potentiometric method. The fitting of the pH-potentiometric data often returns some, seemingly reliable data as a result of multiple deprotonation steps occurring in the pH-range of 1.75–5.00. Stability constants, obtained by simultaneous fitting of the combined data obtained by multiple methods, return more reliable data and are now available for the Cu^2+^ complexes formed with DO3A^3−^, DOTA^4−^, and DO3A-AE^3−^ systems (Table 2). By comparing our results based on the series of spectra shown in Figure 1 (data corresponding to the Cu(DO3A-BACE)^2−^ complex are shown in Figure S3) with the stability data of DO3A^3−^, DOTA^4−^, and DO3A-AE^3−^ complexes one can conclude that they follow the trend which can be predicted based on the basicity of the ligands (Table 1). To overcome the differences observed as a result of the use of different experimental setups (ionic strengths, basicity of the ligands, etc.) we have calculated the pCu values for the Cu^2+^ complexes which are also shown in Table 2. These data indicate that the Cu(DO3A-BACE)^2−^ has the highest conditional stability among the systems and is compared in Table 2.

**Table 2:** Stability constants of DO3A-ACE^4−^, DO3A-BACE^4−^, DOTA^4−^, DO3A^3−^, and DO3A-Nprop^4−^ with the essential alkaline earth (Mg^2+^ and Ca^2+^) and transition metal (Cu^2+^ and Zn^2+^) ions (25 °C).

	DO3A-ACE^4− a^	DO3A-BACE^4− a^	DOTA^4−^	DO3A-*N*prop^4− g^	DO3A-AE^4−h^	DO3A^3− d^
log *K*_CaL_	11.54 (5)	10.29 (2)	16.37 ^b,^ 17.23 ^c^; 16.11 ^f^	12.61	14.00	11.74; 11.35 ^e^; 12.57 ^f^
log *K*_CaL·H_	7.58 (3)	5.00 (8)	3.60 ^b^; 3.54 ^c;^ - ^f^	5.63	7.44	4.60 ^f^
log *K*_CaHL·H_	5.97 (6)	-		4.70	-	-
log *K*_MgL_	7.99 (4)	8.27 (2) ^a^	11.92 ^c^; 11.43 ^f^	12.15	-	9.79 ^e^; 11.64 ^f^
log *K*_MgL·H_	8.84 (3)	-	4.09 ^c^; - ^f^	6.93	-	-
log *K*_MgHL·H_	7.21 (5)	-	-^c^	4.95	-	-
log *K*_ZnL_	19.50 (5)	17.48 (1)	18.70 ^b^; 21.10 ^c^; 20.21 ^f^	17.91	21.45	19.26; 21.57 ^f^
log *K*_ZnL·H_	6.87 (3)	3.82 (1)	5.33 ^b^, 4.18 ^c;^ - ^f^	4.63	7.48	3.77, 3.47 ^f^
log *K*_ZnHL·H_	3.68 (3)	3.07 (1)	3.96 ^b–f^	3.65	4.10	2.07 ^f^
log *K*_ZnH2L·H_	3.14 (2)	1.94 (3)	-	-	2.14	-
log *K*_CuL_	23.47 (10)	24.64 (10)	22.72 ^b^; 22.25 ^c^; 24.83 ^f^	19.64	25.36	22.87, 25.75 ^f^
log *K*_CuL·H_	6.90 (7)	3.31 (6)	4.45 ^b^; 3.78 ^c;^ - ^f^	4.42	7.35	3.2; 3.65 ^f^
log *K*_CuHL·H_	3.98 (4)	3.13 (7)	3.92 ^b;^ - ^f^	4.05	3.67	2.8; 1.69 ^f^
log *K*_CuH2L·H_	1.67 (4)	1.10 (7)	-	-	1.41	-
*p*Cu	18.78	22.41	19.33	15.10	17.11	20.02

^a^ I = 0.15 M NaCl the log *K*_ML·H_ constants shown correspond to the protonation constants of the complexes (log *K*_ML·H_ = [ML·H]/[ML][H^+^]), this work; ^b^ from Ref. [27], I = 0.1 M KCl; ^c^ Ref. [34], 25 °C, I = 0.1 M KNO_3_; ^d^ Ref. [33], I = 0.1 M Me_4_NCl; ^e^ Ref. [35], I = 0.1 M Me_4_NCl; ^f^ Ref. [36], I = 0.1 M KCl; ^g^ Ref. [19], I = 0.1 M KCl; ^h^ Ref. [32], I = 0.1 M KCl; pCu calculated by using the following conditions: c_L_ = 10 μM, c_Cu_ = 1 μM, and pH = 7.4.

**Table 3:** Stability constants of DO3A-ACE^4−^, DO3A-BACE^4−^, DOTA^4−^, DO3A^3−^, and DO3A-Nprop^4−^ with some Ln^3+^ ions (25 °C).

	DO3A-ACE^4−^	DO3A-BACE^4−^	DOTA^4−^	DO3A-*N*prop^4−^	DO3A-AE^3−, k^	DO3A^3−^
log *K*_CeL_	18.13 (9) ^b^	16.99 (17) ^a^; 17.11 (3) ^b^	23.4 ^d^; 21.6 ^f^; 24.6 ^g^	-	20.23 (La^3+^)	19.7^l^; 18.63 (La^3+^) ^m^
log *K*_CeL·H_	6.23 (07) ^b^	-	1.9 ^g^	-	6.64 (La^3+^)	1.25 ^l^
log *K*_GdL_	19.02 (5) ^c^	17.87 (6) ^a^; 18.20 (5) ^c^	24.7 ^d^; 25.3 ^e^; 23.6 ^f^; 24.0 ^h^; 21.1 ^i^	17.91(Zn^2+^) ^j^	22.40	21.0 ^l^; 21.56 ^m^; 19.05 ^n^
log *K*_GdL·H_	8.03 (3) ^c^	3.18 (6) ^a^	2.3 ^h^	-	5.91	2.06 ^l^
log *K*_YbL_	-	18.07 (4) ^a^	25.0 ^d^; 24.0 ^f^; 26.4 ^g^	-	22.09 (Lu^3+^)	21.44 (Lu^3+^) ^m^
log *K*_YbL·H_	-	3.22 (3) ^a^	1.5 ^g^	-	6.53 (Lu^3+^)	-

^a^ This work, I = 0.15 M NaCl, pH potentiometry the log *K*_ML·H_ constants shown correspond to the protonation constants of the complexes (log *K*_LnL·H_ = [LnL·H]/[LnL][H^+^]); ^b^ this work, I = 0.15 M NaCl, UV-Vis; ^c^ this work, 0.15 M NaCl, relaxometry; ^d^ Ref. [38], I = 0.1 M KCl, UV-Vis; ^e^ Ref. [31], I = 0.1 M Me_4_NCl, UV-Vis; ^f^ Ref. [39], I = 1.0 M NaCl, UV-Vis (37 °C); ^g^ Ref. [26], I = 0.1 M Et_4_NCl, 25 °C ; ^h^ Ref. [35], I = 0.1 M Me_4_NCl; ^i^ Ref. [40], I = 1.0 M NaCl, kinetics (UV-Vis, ^153^Gd radiotracer); ^j^ shown as an indicator data, as the stability of Ln(DO3A-Nprop)^−^ complexes were not evaluated in Ref. [19]; ^k^ Ref. [32], I = 0.1 M KCl; ^l^ Ref. [41], I = 0.1 M Me_4_NCl, UV-Vis; ^m^ Ref. [36], 25 °C, I = 0.1 M KCl; ^n^ Ref. [42], I = 0.15 M NaCl.

The stability of the Zn^2+^ complex of DO3A-BACE^4−^ is between that of DOTA^4−^ and DO3A-Nprop^4−^, which is only slightly more stable than that of DO3A-ACE^4−^. In contrast, the Ca^2+^ and Mg^2+^ complexes of DO3A-ACE^4−^ and DO3A-BACE^4−^ are much less stable than those of DOTA^4−^ and DO3A-Nprop^4−^.

These complexes of DO3A-ACE^4−^ and DO3A-BACE^4−^ undergo one or more protonation steps, whose constants are also shown in Table 2. The complexes of DO3A-ACE^4−^ with the four divalent cations studied have a quite high first protonation constant (in the range of 6.87 to 8.84), which can be associated to protonation of the free primary amino group present in the ligand. Such a high protonation constant is not present in the complexes of DO3A-BACE^4−^, as well as of DOTA^4−^, DO3A-Nprop^4−^, and DO3A^3−^. The Cu^2+^ and Zn^2+^ complexes show two protonation constants in the range of 1.7 to 4.4, which can be assigned to protonation of two free carboxylate oxygens in trans position, which were also observed in the DOTA^4−^ and DO3A-Nprop^4−^ complexes.

The stability constants of the complexes of the DO3A-ACE^4−^ and DO3A-BACE^4−^ ligands with the lanthanide(III) ions Ce^3+^, Gd^3+^, and Yb^3+^ are obtained by potentiometric titrations. The stability constants of the Ce^3+^ and Gd^3+^ complexes are also obtained by UV-Vis spectrophotometry and water proton *T*_1_ relaxometry, respectively. Their values are listed in Table 3, and compared with the corresponding values available in the literature for the DOTA^4−^, DO3A^3−^, and DO3A-Nprop^4−^ complexes [26,31,35,36,38,39,40,41,42]. The stability of the complexes with DO3A-ACE^4−^ and DO3A-BACE^4−^ increases as the Ln^3+^ ionic radius decreases along the lanthanide series, in accordance with the trend observed for DOTA^4−^ and DO3A^3−^. The stability constants of the different ligands with the same Ln^3+^ ion (e.g., Gd^3+^) increase in the order DO3A-BACE^4−^ ≈ DO3A-Nprop^4−^ < DO3A-ACE^4−^ < DO3A^3−^ < DOTA^4−^, which does not obey the stability order expected from the increase of the ligand basicity expressed by the values of the sum of their first four protonation constants involving their macrocyclic N and carboxylate O atoms (Σ log *K*_i_^H^, i = 1–4, Table 1) [43], except for the relative values of DO3A^3−^ < DOTA^4−^, which indicates the presence of steric effects in some of the complexes. The large decrease of the log *K* values of the DO3A-BACE^4−^ and DO3A-ACE^4−^ Gd^3+^ complexes relative to DOTA^4−^ (and even DO3A^3−^) results, in part, from the lower stability of the six-membered chelate ring relative to a five-membered chelate ring formed between the Gd^3+^ and the propionate vs. the acetate pendant arm as observed before for DO3A-Nprop^4−^ [19], which cause a larger destabilization of the complexes than the absence of one acetate pendant arm in Gd(DO3A). The presence of an α-amino group in the propionate arm in DO3A-ACE^4−^ stabilizes the complex relative to DO3A-Nprop^4−^, but the α-benzylamide group has no adverse effect on the complex stability. Similar to the case of the complexes with alkaline earth and transition metal ions, there is a quite high protonation constant in the Ce(DO3A-ACE)^−^ and Gd(DO3A-ACE)^−^ complexes (6.23 and 8.03, respectively), associated with protonation of the free amino group of the ligand. This is much higher than the values for the Gd(DO3A-BACE)^−^ (3.18), Gd(DO3A) (2.06), and Gd(DOTA)^−^ (2.3) complexes, which likely correspond to protonation of carboxylate oxygens.

### 2.2. Formation Kinetics of Ln(DO3A-ACE)^−^ Complexes

One of the ideas driving the design and synthesis of the DO3A-*N*prop^4−^ ligand is the fact that its Gd^3+^ complex possesses a considerably fast water exchange rate and this is expected to influence the formation rate of its Ln^3+^ complexes. In fact, the Gd(DO3A-*N*prop)^−^ complex is formed somewhat faster than the Gd^3+^ complex formed with the DOTA ligand, although the formation rate increases less than expected [19]. Thus, we have decided to look at the formation kinetics of the Eu(DO3A-ACE)^−^ complex in order to obtain data on how the amine functionality affects the rate of formation of the Eu^3+^ chelate. In the pH range studied (4.51 to 5.41), the formation of the complex is slow enough to be followed using UV-Vis spectrophotometry by following changes in the Eu^3+^ charge transfer (CT) bands (broad “shoulder” between 240 and 305 nm). Λ = 255 nm is selected for these studies since, at this wavelength (Figure S4) the absorbance of Eu^3+^ and ligand are not significant and the Eu(DO3A-ACE)^−^ complex is the only absorbing species. 

**Figure 2:**
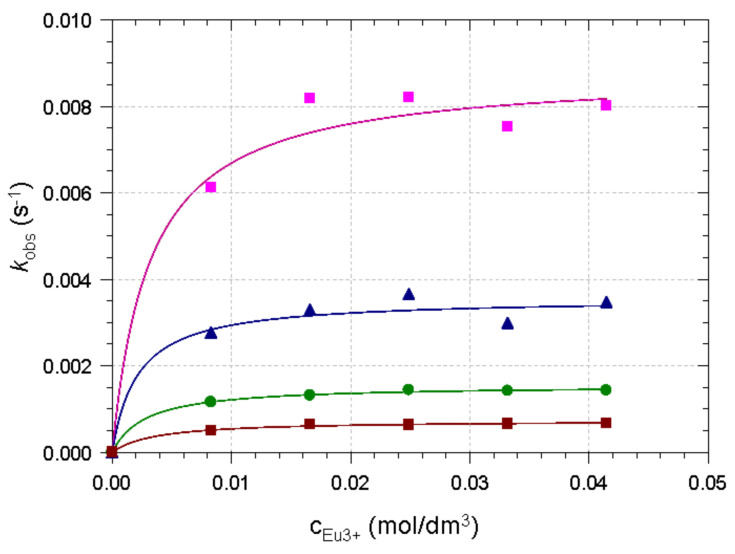
Pseudo-first-order rate constants (k_obs_) as a function of the Eu^3+^ concentration (c_Eu3+_) in the formation reaction of Eu(DO3A-ACE)^−^: pH = 5.41 (■); 4.82 (▲); 5.11 (●); 4.51 (■) (c_L_ = 1.60 mM, 25 °C: 0.15 M NaCl). The solid lines represent the simultaneous fit of the data at all pH values to Equation (3).

In the presence of Eu^3+^ excess (pseudo-first-order conditions) the formation rate of the complex can be expressed according to Equation (1):

(1) $$\frac{d{\left[\mathrm{LnL}\right]}_{t}}{\mathrm{dt}}=k_{\mathrm{obs}}\;{\left[L\right]}_{t}$$

where [L]_t_ is the total concentration of the free DO3A-ACE^4−^ ligand and k_obs_ is the pseudo-first-order rate constant. The formation reactions have been investigated at different pH values and Eu^3+^ concentrations. Plots of k_obs_ values against metal concentration give saturation curves, for all Eu^3+^ and pH values used (Figure 2). Such a saturation behavior has been previously observed for other Ln^3+^ macrocyclic complexes, which is explained by the rapid formation of an intermediate that rearranges in a slow, rate determining step [18,19,44,45]. According to previous results on DOTA-type complexes, the intermediate is assumed to be triprotonated (evidenced in the absorption spectra of the Ce^3+^ complex as shown in Figure S5), two protons at the macrocyclic nitrogens and one at the primary amine of the ACE arm, H_3_L*, with a stability constant:

(2) $$K^{*}{}_{{\mathrm{LnH}}_{3}L}=\frac{\;\left[{\mathrm{LnH}}_{3}L^{*}\right]}{\left[\mathrm{Ln}\right]\left[H_{3}L\right]\;}$$

As the concentration of the fully deprotonated and monoprotonated ligand (at the macrocyclic nitrogen) is practically zero at the pH values of the complex formation study, the value of *k*_obs_ can be derived to be:

(3) $$k_{\mathrm{obs}}=\frac{k_{r}\left(K_{\mathrm{Ln}{\left(H_{3}L\right)}^{3+}}/\alpha_{H}\right)\left[{\mathrm{Ln}}^{3+}\right]}{1+\left(K_{\mathrm{Ln}{\left(H_{3}L\right)}^{3+}}/\alpha_{H}\right)\left[{\mathrm{Ln}}^{3+}\right]}$$

where *k*_r_ is the rate constant of the deprotonation and rearrangement of the intermediate to the product and α_H_ is the fraction of free L in H_3_L form [18,19,44,45].

The pseudo-first-order rate constants at the various pH values and metal concentrations (Figure 2) have been simultaneously fitted to Equation (3), and the rate constants, *k*_r_, and the stability constant of the intermediate, *K*_LnH3L*_, are calculated using the ligand protonation constants and *k*_r_ is found to be 4.2 × 10^2^ (s^−1^) (corresponding to log *K*_LnH3L*_, of 2.61 (0.08)) The given value is larger than those observed for Ln(DO2A)^+^ complexes (in the range of 1.98 (Ce^3+^)–1.60 (Yb^3+^)) or those formed with the DO2A2M^nBu2−^ ligand possessing two acetate and two amide functionalities (2.66 (Ce^3+^); 2.47 (Eu^3+^) and 1.67 (Yb^3+^)) [46,47]. On the other hand, the given value is smaller than the corresponding values determined for the complexes of aminoethyl-DO3A (DO3A-AE^3−^) synthesized recently as pH responsive MRI CA candidates (4.91 (La^3+^), 4.89 (Gd^3+^) and 4.44 (Lu^3+^)) [32]. Based on these data, one can assume that at least two acetate pendant arms are involved in the coordination of the Eu^3+^ ion in the given intermediate. Very likely, the arm possessing the positively charged amine functionality remains uncoordinated in the intermediate complex.

**Figure 3:**
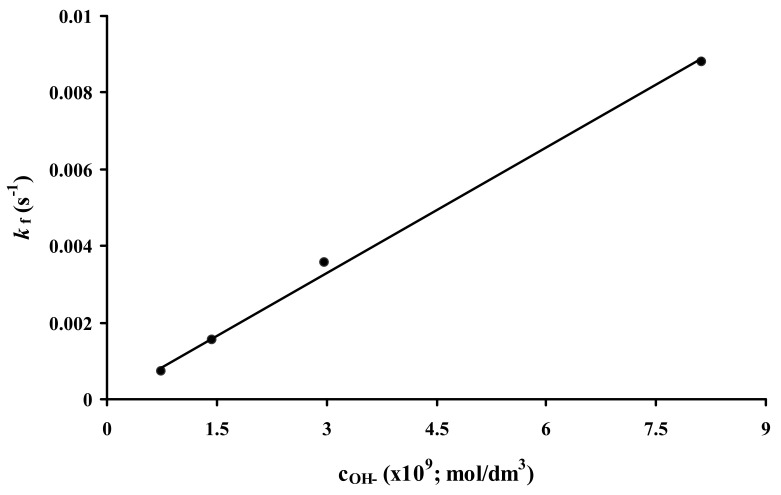
Formation rate constants, k_f_ (s^−1^), as a function of OH^−^ concentration for the Eu(DO3A-ACE)^−^ complex (25 °C).

In order to complete the reaction, the given intermediate must lose two protons and rearrange to the final complex through a general base catalyzed reaction (the catalyst is OH^–^ or any other base present in the sample). The rate constants *k*_f_ characterizing the formation rates of the Eu^3+^ complex at different pH values are presented in Figure 3 as a function of OH^–^ concentration. The data in Figure 3 show that the *k*_f_ rate constants are inversely proportional to the proton concentration and directly proportional to the OH^–^ concentration, i.e., *k*_f_ = *k’*/[H^+^] = *k*_OH_ [OH^–^] (where *k*_OH_ = *k*’/*K*_w_). The linear increase of the formation rates of the complex with increasing OH^–^ concentration suggests a OH^–^ catalyzed deprotonation of the Ln(H_3_L) intermediate, but the interpretation of experimental data is somewhat more complicated. Detailed studies have shown that a dissociation equilibrium exists between the Ln(H_3_L) and Ln(H_2_L) intermediates (Ln(H_3_L) ⇌ Ln(H_2_L) + H^+^). It is well known from the literature [26] that the rate determining step of complexation is the loss of the proton from the monoprotonated intermediate. However, the concentration of the Ln(H_3_L) intermediate is directly proportional to 1/[H^+^] due to the dissociation equilibrium. The 1/[H^+^] dependence of the formation rates can be simply described as dependence on the OH^–^ concentration, so the k_OH_ rate constants can be conveniently used for comparison. This very simple rate law is similar to those observed for the complex formation reactions of a large variety of tetraazacyclododecane, as well as cyclotridecane macrocyclic ligand derivatives [19,43,48,49,50,51,52,53].

The calculated k_OH_ values are listed in Table 4, where some data for the formation of other DOTA derivative complexes are also shown for comparison. The data collected in Table 4 indicate that the replacement of an acetate pendant arm in DOTA^4−^ by the 1-(2-amino-carboxyethyl) group results in a considerable (an order of magnitude) decrease in the formation rates of its Eu^3+^ complex, which is likely the result of the presence of protonated and thus positively charged amine functionality generating a greater repulsion between the ligand and positively charged Eu^3+^ ion. Similar results were found recently for aminoethyl-DO3A systems (DO3A-AE^3−^ and DO3A-DMAE^3−^) synthesized as pH responsive *T*_1_-MRI CA candidates [34]. 

### 2.3. Dissociation Kinetics of Ln^3+^ Complexes

**Scheme 2:**
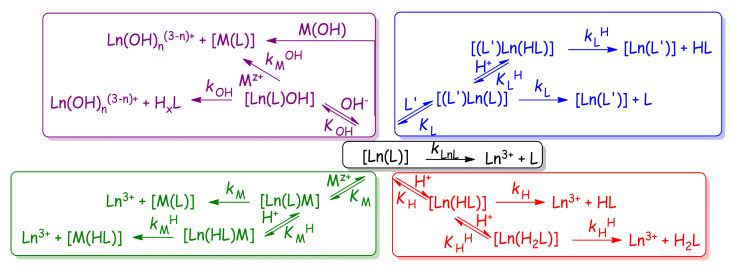
Possible dissociation pathways of Ln^3+^ complexes.

The kinetic inertness of Ln^3+^ complexes is a very important factor of their in vivo safety. The main pathways, through which the decomposition of a LnL complex can occur, are shown in Scheme 2, which depend mainly on the structure of the ligand L and on the solution pH. The spontaneous dissociation path (shown in black in Scheme 2) is important at pH close to neutral, while at lower pH, proton-assisted pathways involving the formation of mono- and deprotonated complexes predominate (shown in red in Scheme 2). Pathways involving the catalysis by the most abundant endogenous metal ions, Cu^2+^ or Zn^2+^, can also occur, although they are less likely for the macrocyclic complexes, and the metal-assisted dissociation depends on the stability of dinuclear Gd(L)Cu or Gd(L)Zn complexes (shown in green in Scheme 2). Ligand exchange reactions (shown in blue in Scheme 2) usually do not contribute to dissociation of GdL complexes of macrocyclic ligands (since the formation of ternary complexes L´GdL is very unlikely) yet the given dissociation path was found to be important recently for Ln^3+^ complexes of DTPA and DTPA-bis(amide) derivatives. Moreover, hydroxide-assisted dissociation pathways (shown in lilac in Scheme 2)) do not contribute to the dissociation at neutral or slightly acidic pH values, whereas the given pathway adds an important contribution for Ga^3+^ complexes [2,18,19,45,54,55]. 

The kinetic inertness of the Ce^3+^ and Gd^3+^ complexes of DO3A-ACE^4−^ and DO3A-BACE^4−^ was evaluated at 25 °C through the study of their dissociation kinetics in acidic conditions, with a constant total ionic strength I (HCl + NaCl) = 1 M. The proton-assisted dissociation of the Ce^3+^ complexes was investigated by the UV-Vis method, while the same study for the Gd^3+^ complexes, as well as the exchange reaction of Gd(DO3A-BACE)^−^ complex occurring with Zn^2+^, were carried out by water proton *T*_1_ relaxometry. 

**Figure 4:**
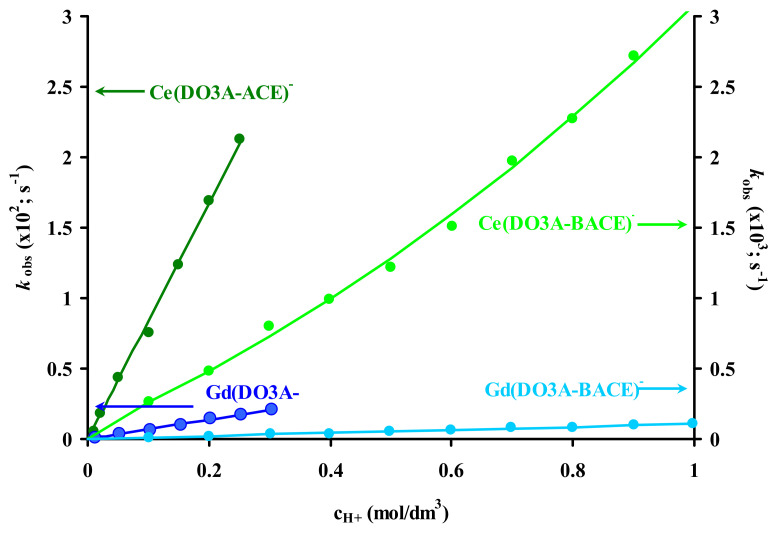
Dependence of the dissociation rate constants (*k*_obs_) on the H^+^ concentration in the dissociation of Ce(DO3A-ACE)^−^ (green), Gd(DO3A-ACE)^−^ (blue), Ce(DO3A-BACE)^−^ (light green), and Gd(DO3A-BACE)^−^ (light blue). The lines correspond to the fit according to Equations (5) and (6).

In the presence of a large excess of HCl, the complexes are thermodynamically unstable and dissociate completely. Under these conditions, the reaction occurs with a rate which is proportional to the total concentration of the LnL complex:

(4) $$\frac{d{\left[\mathrm{LnL}\right]}_{t}}{\mathrm{dt}}=k_{\mathrm{obs}}\;{\left[\mathrm{LnL}\right]}_{t}$$

where *k*_obs_ is the pseudo-first-order rate constant and [LnL]_t_ is the total concentration of the complex (under the condition applied it is equal to [LnL] + [LnHL] + [LnH_2_L] + [LnLM], the last term applicable only for the metal exchange reactions). Figure 4 shows the dependence of dissociation rate constants (*k*_obs_) with [H^+^], obtained in the H^+^ ion concentration range of 0.025–0.25 M (for Ln(DO3A-ACE)^−^) and 0.1–1.0 M for Ln(DO3A-BACE)^−^). The plots of k_obs_ as a function of proton concentration indicate that the Ln^3+^ complexes of DO3A-ACE^4−^ undergo faster dissociation in acidic conditions than their DO3A-BACE^4−^ counterparts. This can be rationalized by the presence of the amino group which protonates readily by forming quantitatively LnHL complexes below pH = 5.0 and likely contributes to the efficient transfer of these protons to the macrocyclic nitrogens required for the dissociation to occur. This, in fact, means that the parent compound, possessing the amine nitrogen required for bioconjugation purposes, is considerably less inert than the one (DO3A-BACE^4−^) modelling the coordination environment of the complex conjugated to a biological vector.

The plots of *k*_obs_ versus H^+^ concentration obtained for the Ln^3+^ complexes of DO3A-ACE^4−^ and DO3A-BACE^4−^ chelators present rather different shapes (Figure 4). For the complexes of DO3A-ACE^4−^ the dissociation rates show a linear behavior with proton concentration, while for DO3A-BACE^4−^ a second order dependence is observed. The [H^+^] dependence of *k*_obs_ can be described by the sum of the contributions from three possible dissociation pathways: The spontaneous dissociation of the LnL complex, with rate constant *k*_0_ (being equal to *k*_LnL_ in Scheme 2), the proton-assisted pathway involving dissociation of the monoprotonated LnHL complex (characterized with protonation *K*_H_ and rate constant *k*_H_), and dissociation of the diprotonated LnH_2_L complex, with protonation *K*_H_^H^ and rate constant *k*_H_^H^. These contributions are described by Equation (5). In all cases, the observed intercept is nearly zero, indicating the absence of spontaneous dissociation (*k*_0_ being equal to *k*_LnL_ in Scheme 2). The linear dependence of DO3A-ACE^4−^ complexes can be attributed to the rapid formation of a stable monoprotonated intermediate (characterized with protonation constants *K*_H_ and rate constant *k*_1_, where *k*_1_ = *k*_H_·*K*_H_) quantitatively at the beginning of the reaction. Thus, the observed first-order dissociation rates are analyzed using Equation (5). For the complexes of DO3A-BACE^4−^, the *k*_obs_ values are analyzed using the Equation (6) expression:

(5) $$k_{\mathrm{obs}}=k_{0}+k_{1}\left[H^{+}\right]$$

and

(6) $$k_{\mathrm{obs}}=k_{0}+k_{1}\left[H^{+}\right]+k_{2}{\left[H^{+}\right]}^{2}$$

In this equation, *k*_2_ (where *k*_2_ = *k*_H_^H^·*K*_H_·*K*_H_^H^) represents the rate constant characterizing the dissociation of the complex with the formation of a diprotonated species. Similar dependencies with proton concentration are observed for Ln^3+^ complexes with a cyclen-based ligand containing a picolinate pendant [50], as well as that of Ce(DOTA)^−^ [56]. The rate constants determined using these equations are listed and compared to those obtained for the complexes of similar ligands in Table 5.

Since the dissociation of Ln(DO3A-ACE)^−^ complexes occurs considerably faster than those formed with macrocyclic ligands of similar size, we attempted to study metal exchange reactions occurring with Zn^2+^ ions (in the pH range of 3.96–5.32 using 10-, 20-, 30- and 40-fold excess of the exchanging metal ion). The dependence of the decomposition rate constants (*k*_obs_) on [H^+^] and [Zn^2+^] for the Gd(DO3A-ACE)^−^ complex is shown in Figure S6. The *k*_obs_ values increase with [H^+^] at a given Zn^2+^ concentration, but they decrease with the increasing Zn^2+^ concentration at constant pH. This can be explained by the formation of a dinuclear complex Gd(L)Zn which cannot dissociate directly (i.e., dead-end complex), as observed before for macrocyclic complexes such as Gd(TRITA)^−^ [18]. The formation of the intermediate occurs due to the presence of the noncoordinating (to the Gd^3+^ ion) amino group, yet its formation seemingly is not enough to activate (weaken) considerably the complex and therefore, the direct attack of Zn^2+^ on the complex (observed often for Gd^3+^ complexes with DTPA-derivatives) does not occur [19]. The reaction becomes slower as the concentration of the protonated complex decreases as a result of the formation of the dinuclear Gd(L)Zn complex.

Taking all possible pathways into account and equilibrium, as well as rate constants characterizing various pathways, the rate of metal exchange can be expressed as shown in Equation (7) [18,19,57]:

(7) $$\;\;\;\;\;\;\;k_{obs}=\frac{k_{0}+k_{1}\left[H^{+}\right]+k_{3}\left[{\mathrm{Zn}}^{2+}\right]}{1+K_{H}\left[H^{+}\right]+K_{M}\left[{\mathrm{Zn}}^{2+}\right]}$$

where *k*_3_ is the rate constant (where *k*_3_ = *k*_M_·*K*_M_) for the metal-assisted dissociation pathway, *K*_H_ is the protonation constant of the GdL complex, and *K*_M_ is the stability constant of the dinuclear intermediate complex Gd(L)Zn. The *k*_obs_ data determined are included in the supporting information (Figure S6), while the rate constants characterizing spontaneous, acid and metal ions assisted dissociation obtained by the fitting of experimentally determined rate constants to Equation (7) are also shown in Table 5. By analyzing the data listed in Table 5, one can conclude that the Ln^3+^ complexes of the DO3A-ACE^4−^ and DO3A-BACE^4−^ ligands possess an inertness that lies between the data obtained for the DO3A^3−^ and DOTA^4−^ complexes. Similar results were found for the Ce(DO3A-*N*prop)^−^ by É. Tóth et al., which indicate that the inertness of Ln^3+^ complexes drops with the introduction of propionate pendant arm in lieu of acetates. This process is even more pronounced when the acetate group is replaced by the aminopropionate group in DO3A-ACE^4−^, which is likely the consequence of the presence of the amino group acting as a “dock” (site) for the proton attacking the complex. This is in agreement with the experimental finding of the relatively stable heterodinuclear intermediate formation while studying the metal exchange reactions for the Gd(DO3A-ACE)^−^ complex. This trend, however, was reversed for the Ln(DO3A-BACE)^−^ complexes, as by eradicating the protonation site, by converting the amino group into the amide mimicking the bioconjugation, the inertness of the corresponding complexes improved considerably. These results are promising, as they indicate that the DO3A-ACE^4−^ bifunctional ligand, upon its conjugation to vector molecules, such as peptides, affibodies, monoclonal antibodies, nanoplatforms, etc., is expected to offer a coordination cage forming inert Ln^3+^ complexes applicable in vivo.

### 2.4. Dynamic NMR Studies of the Ln(DO3A-ACE)^−^ and Ln(DO3A-BACE)^−^ Complexes

The structure and molecular dynamics of Ln(DOTA)-type chelates in the solution are well known [58,59,60,61]. There are four possible stereoisomeric structures for these chelates that are related as two enantiomeric pairs. Each structure has a stereochemistry defined by two elements of helicity: The conformation of the macrocyclic ring (either *δδδδ* or *λλλλ*) and the orientation of the pendant arms (either *Δ* or *Λ*). They combine into two stereoisomeric pairs of enantiomers: *Λ*(*δδδδ*) ⇌ *Δ*(*λλλλ*), with opposite helicity of the ring and the arms, leading to the SA coordination, and *Δ*(*δδδδ*) ⇌ *Λ*(*λλλλ*), with the same ring and acetate helicity, which give the TSA structure. These can interconvert in the solution by either ring inversion ((*δδδδ*) ⇌ (*λλλλ*)) or acetate arm rotation (*Δ* ⇌ *Λ*). When both processes are combined, either in succession or concerted, there is an exchange between enantiomeric pairs. These intramolecular conformational exchange processes, with rate constants of the order of 10 s^−1^, are relatively slow on the NMR-time scale [61], allowing separate detection of each coordination geometry in the ^1^H NMR spectrum of a chelate. Analysis of variable temperature ^1^H EXSY spectra of Yb(DOTA)^−^ has shown that the rates of arm rotation and ring inversion processes are very similar, suggesting a concerted enantiomerization mechanism, while qualitative studies point to a faster arm rotation than ring inversion [59,62]. All results reflect the high rigidity of the Ln^3+^ complexes of DOTA^4−^.

Substitution of a DOTA^4−^ chelator has a strong effect on the number of isomers present and on their exchange mechanisms. Introducing a chiral carbon center into the ligand framework turns the four stereoisomeric structures described above into diastereoisomers. However, the introduction of a single chiral center in DOTA^4−^ by derivatizing one of the acetate α-carbons sterically locks the conformation of all the pendant arms, whose orientation is determined by the configuration at the C_α_: An R configuration generates *Λ* orientation, while an S configuration leads to the *Δ* orientation. This results from the minimization of the torsional strain of the chelate, where the stereochemical control over the orientation of the substituted pendant arm by placing the bulky substituent anti- to the metal ion with respect to the C-N bond leads to maximization of the number of pendant arms that cooperatively adopt a lower energy conformation with the same helicity [63]. This mechanism leads to the presence of only two species in the ^1^H NMR spectrum of Yb(*p*-NB-DOTA)^−^ (*p*-NB = *para*-nitrobenzyl) [64] Ln(DOTASA) [24,65] and Yb(HP-DO3A) (structures appearing in the NMR part are included in ESI in Figure S7) [66], indicating that two of the four structures are energetically inaccessible. ^1^H EXSY and ROESY spectra showed that the isomers interconvert by fast ring inversion, which is faster than the substituted pendant arm rotation, which is faster than the acetate arms rotation.

This stereochemical control is also observed in the Ln(TCE-DOTA) [52,63] and Ln(*RRRR*-DOTMA)^−^ [67,68] chelates with the four C_α_ substituted acetate arms, where only two species are observed in their ^1^H NMR spectra. The conformation of the macrocycle may also be locked in a single conformation by ring substitution. A single methyl ring substituent, such as in the MDOTA^4−^ ligand, is enough to rigidify the tetraaza macrocycle but not the acetate arms, as shown by the ^1^H spectrum of the racemic (*R/S*) Yb(MDOTA)^−^, with four totally asymmetric isomers forming two enantiomeric pairs, with SA or TSA geometries, for each (*R* or *S*) orientation of the methyl group [69]. Ln(*p*-NB-DOTA)^−^, with a single *p*-NB ring substituent, also shows two isomers in the solution [70]. The Yb(M4DOTA)^−^ complex, where the ligand has four ring methyl substituents with S configuration, originates three species in the solution detected by ^1^H NMR. The methyl substituents again prevent ring inversion but not acetate arms rotation, and the EXSY spectrum shows an exchange between two of the forms via fast acetate rotation [69]. However, Yb(M4DOTMA)^−^, where the ligand has four ring methyl substituents and four C_α_ methyl substituted acetate arms, is a totally rigid complex with a SA geometry [69].

The Ln(DO3A-ACE)^−^ and Ln(DO3A-BACE)^−^ complexes have a single chiral center at their substituted acetate α-carbon, which is racemic as a consequence of the synthesis of the ligands [20,22]. The ^1^H NMR spectrum of the paramagnetic Eu(DO3A-ACE)^−^ complex in the solution corresponds to a dominant SA isomer conformation with C_1_ symmetry, with a residual (2%) presence of the TSA isomer [20]. As the SA isomer population is expected to increase for the complexes of the smaller Y^3+^ cation relative to the Eu^3+^ complex [59,60], the population of the TSA isomer can be assumed to be negligible for the Y^3+^ complexes.

**Figure 5:**
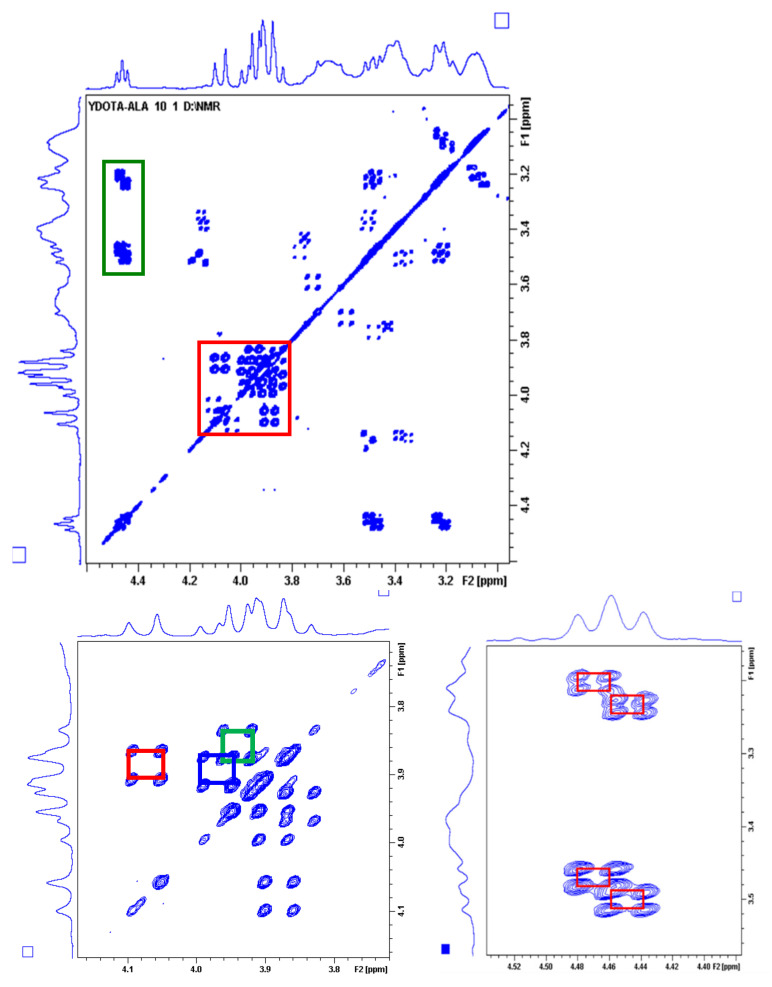
^1^H 2D COSY spectrum of Y(DO3A-ACE)^−^ in D_2_O at 80 °C (**top**) and two expansions (**bottom**): A2, A3, and A4 arm protons are in the left, while the cross peaks between A1s and A1ab protons are in the right.

**Scheme 3:**
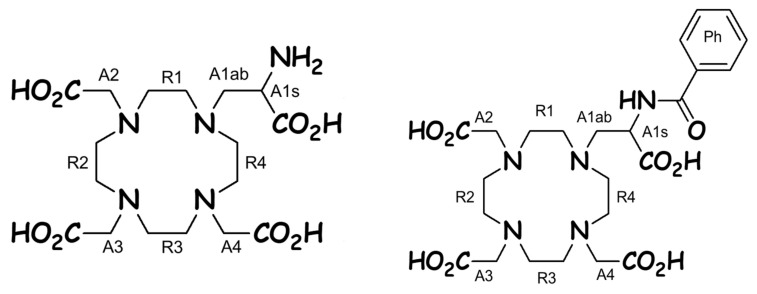
Chemical structures of the H_4_DO3A-ACE and H_4_DO3A-BACE ligands and numbering scheme of their protons.

**Figure 6:**
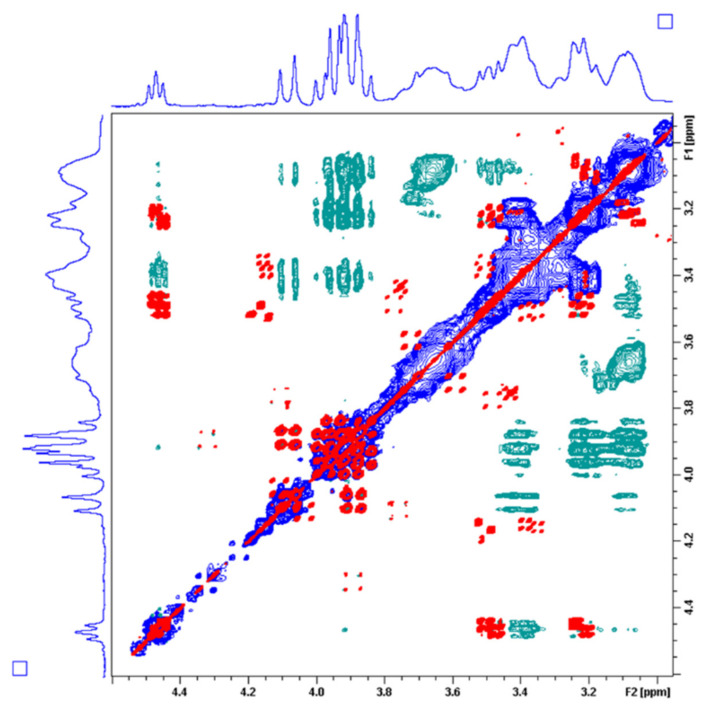
Overlap of the ^1^H 2D COSY (red) and NOESY spectra of Y(DO3A-ACE)^−^ in D_2_O at 80 °C. NOESY blue cross peaks are in the same phase of the diagonal and green are in the opposite phase.

**Scheme 4:**
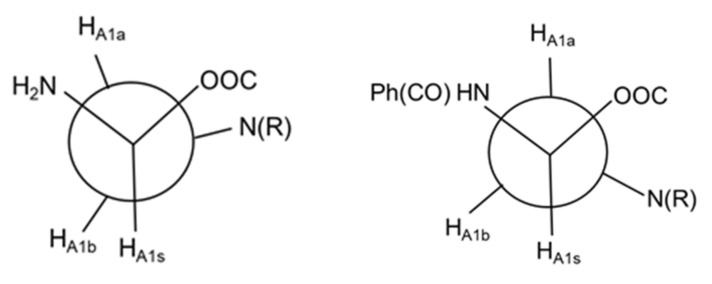
Newman-projections of conformation along the C_1ab_-C_1s_ bond of the Cα-substituted pendant arm of Y(DO3A-ACE)^−^ (**left**) and Y(DO3A-BACE)^−^ (**right**).

The diamagnetic Y(DO3A-ACE)^−^ complex shows a temperature dependent ^1^H NMR spectrum, which is quite broad at low temperature (0–47 °C), and becomes sharper at high temperatures (57 to 80 °C) (Figure S8). This reflects an intramolecular dynamic isomerization process of the complex between the SA pair of enantiomers, R-*Λ*(*λλλλ*) and S-*Δ*(*δδδδ*). At 80 °C, the 1D spectrum (Figure S8) is sharp and its intensities indicate that the total and relative areas of the different peaks are in agreement with those expected for a chelate of 4-fold symmetry. Assignments are made on the basis of the relative peak areas, their coupling patterns, and the COSY spectrum (Figure 5 top), referred to the proton numbering of Scheme 3. The methylene protons of the three acetate arms (A2, A3, and A4) originate three AB doublets with shifts of 4.08 and 3.88 ppm for A2ab, 3.97, and 3.89 ppm for A3ab and 3.94 and 3.86 ppm for A4ab with the corresponding COSY cross-peaks (Figure 5, bottom left). Another separate spin system is formed by the A1ab methylene protons and the A1s methine proton of the C_α_ substituted arm, forming an ABX spin system. The pseudo triplet at 4.46 ppm corresponds to the A1s proton and the non-equivalent A1ab protons appear at 3.48 and 3.22 ppm, respectively, as shown by the COSY cross-peaks (Figure 6 bottom right). The vicinal coupling constants obtained from spectral simulation are quite similar, ^3^J_As,A1a_ = 7.4 Hz and ^3^J_As,A1b_ = 8.4 Hz, from which the dihedral angles between them of about 30 and 150° were obtained using the Karplus equation [71,72], as illustrated by the corresponding Newman projection of the conformation along the C_1ab_-C_1s_ bond (Scheme 4, left).

The ring protons R_1–4_ cannot be fully resolved even at 80 °C due to the fluxional motion of the ring being in the intermediate exchange regime in the NMR time scale, as illustrated by Figure S8. At low temperatures up to 37 °C, the resonances of the arms are fully broadened out and those from the ring protons are very broad, practically unresolved peaks. This indicates that the fluxional motion of the C_α_-substituted DOTA-type complex Y(DO3A-ACE)^−^ is in the intermediate exchange regime, but the acetate arms rotation, also in intermediate exchange, is faster than the ring inversion process [58,61]. At higher temperatures, the motion of the arms accelerates first, reaching the fast exchange limit at 70 °C, while the ring motions do not reach the fast exchange regime even at 80 °C, remaining broad and showing very few COSY cross-peaks. These slower internal ring motions relative to the arms motions are the opposite of what has been observed for other C_α_-substituted DOTA-type complexes [63,64], which could result from steric hindrance from the amino substituent.

The 2D NOESY spectrum of Y(DO3A-ACE)^−^ at 80 °C (Figure 6 and Figure S10) shows two types of cross-peaks. Those with the same phase of the diagonal peaks (blue) result from magnetization transfer through chemical exchange, and those in opposite phase with the diagonal peaks (green) result from NOE magnetization transfer through dipolar cross-relaxation [73]. Strong exchange cross peaks are observed between the methylene protons of the three acetate arms (but not for the substituted arm), which exchange during the helicity *Δ*/*Λ* change. Some peaks are also present between ring protons, which switch their positions during ring inversion. On the other hand, several NOE peaks are observed between the arm and ring protons, indicating that the coordination moved these protons to be close in space in the complex. Comparing the superimposed COSY-NOESY spectra (Figure 6), it becomes clear that there are cross peaks between the arms and ring protons and between ring protons other than COSY peaks, indicating again formation of a compact ligand structure in the complex. An interesting example is the NOE cross peak of the methine proton A1s with ring protons.

**Figure 7:**
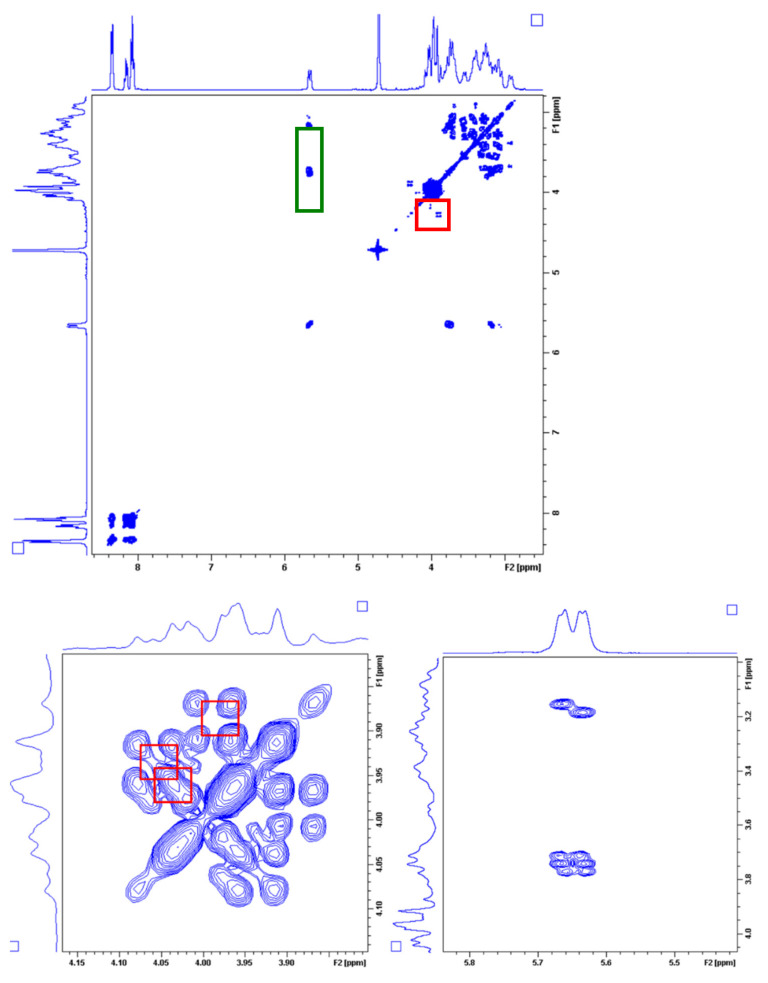
^1^H 2D COSY spectrum of Y(DO3A-BACE)^−^ in D_2_O at 80 °C (**top**) and two expansions (**bottom**): A2, A3, and A4 arm protons are in the left, while the cross peaks between A1s and A1ab protons are in the right.

The ^1^H NMR spectrum of Y(DO3A-BACE)^−^ is also temperature dependent but becomes quite sharp at 80 °C (Figure S11), reflecting similar dynamic processes to those present in Y(DO3A-ACE)^−^. Its assignments are also made on the basis of the relative peak areas, their coupling patterns, and the COSY spectrum (Figure 7 top). The multiplets from the aromatic ring protons are centered at 8.35, 8.18, and 8.10 ppm. There are three AB doublets from the methylene protons of the three acetate arms (A2, A3, and A4), with shifts of 4.08 and 4.03 ppm for A2ab, 4.06 and 4.01 ppm for A3ab, and 4.00 and 3.96 ppm for A4ab, with the respective COSY cross-peaks (Figure 7, bottom left). An ABX spin system with the corresponding COSY cross-peaks can also be seen for the A1ab methylene protons at 3.74 and 3.17 ppm and the A1s methine proton at 5.65 ppm (Figure 7, bottom right). The large upfield shift of the A1s proton results from the ring current effect of the nearby aromatic ring. The A1s is a doublet of doublets due to the very different vicinal coupling constants, ^3^*J*_A1s,A1b_ = 3 Hz and ^3^*J*_A1s,A1a_ = 11.2 Hz, which correspond to dihedral angles of 45 and 180° between them along the C_1ab_-C_1s_ bond. This reflects the changed orientations of the two bulky groups of the side-chain, Ph(CO)NH an N(R), which are as far away from each other as possible, as illustrated by the corresponding Newman projection (Scheme 4, right).

**Figure 8:**
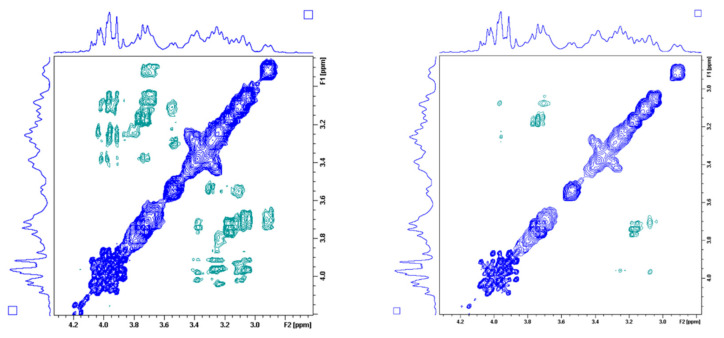
Expanded area of the ^1^H 2D NOESY spectrum of the Y(DO3A-BACE)^−^ complex at 80 °C shown in Figure S13. The left spectrum has an increased intensity relative to the right one. Blue cross peaks are in the same phase as the diagonal, while the green ones are in the opposite phase.

The temperature dependence of the ^1^H NMR spectra of the Y(DO3A-BACE)^−^ complex (Figure S12) indicates that the acetate arm rotation and ring inversion motions are faster at each temperature than for Y(DO3A-ACE)^−^. The proton resonances from the acetate arms are present even at the very low temperatures, although they are as broad as the ring protons. At higher temperatures, the acetate arms rotation and ring inversion become faster and reach the fast exchange limit at 80 °C, showing a large number of COSY cross-peaks. The internal ring motions in Y(DO3A-BACE)^−^ are faster relative to Y(DO3A-ACE)^−^, and are closer to what has been observed for other C_α_-substituted DOTA-type complexes [64]. This could result from the absence of steric hindrance from the bulky amino substituted Ph(CO)NH group, which is rearranged to a position further away from the ring. This is supported by the lack of NOE cross peaks between the aromatic protons and either the ring or the arm protons in the 2D NOESY spectrum (Figure S13). Enlargement of the labeled box of this spectrum (Figure 8) shows similar exchange and NOE cross peaks as for Y(DO3A-ACE)^−^, with exchange peaks between arm protons (except for the substituted one) at the low intensity spectrum (right), and NOE peaks between the arm and ring protons and some between ring protons at the high intensity spectrum (left).

## 3. Materials and Methods

### 3.1. General

DO3A-ACE^4−^ and DO3A-BACE^4−^ were synthesized and purified according to procedures described in the literature [20,22]. Their purity was confirmed by ^1^H, ^13^C NMR as well as EA and ESI-MS analysis.

### 3.2. Preparation of the Stock Solutions

Stock solutions of the MgCl_2_, CaCl_2_, ZnCl_2_, CuCl_2_ YCl_3_, and LnCl_3_ were prepared from analytical grade salts (Aldrich and Sigma, Burlington, MA, USA, 99.9%). The concentrations of the stock solutions were determined by complexometric titration using standardized Na_2_H_2_EDTA (Fluka Analytical, Darlington, UK, 99–101%) solution in the presence of an appropriate end-point indicator (eriochrome black-T for MgCl_2_, and ZnCl_2_, calconcarboxylic acid for CaCl_2_, murexide for CuCl_2_, and xylenol orange for YCl_3_ and LnCl_3_ stock solutions). The concentrations of the ligands were determined by pH-potentiometry, on the basis of titration curves of the ligands obtained in the absence and presence of 10-fold excess of CaCl_2_.

The protonation constants of the ligand were calculated from the data obtained by titrating 2.38 and 3.38 mM ligand solutions (total 189 and 211 data pairs, respectively) with standardized NaOH solution (0.2254 M) in the pH range of 1.78–11.80. The protonation constants of the ligand (log *K*_i_^H^) are defined as:

(8) $$K_{H_{i}L}=\frac{\;\left[H_{i}L\right]}{\left[H^{+}\right]\left[H_{i-1}L\right]\;}$$

where i = 1, 2, …, 6 and [H_i-1_L] and [H^+^] are the equilibrium concentrations of the ligand.

### 3.3. Equilibrium Measurements

The protonation constants of the ligands and the stability constants of the complexes formed with Mg^2+^, Ca^2+^, and Zn^2+^ were determined using direct pH-potentiometric titrations. The determination of the Cu^2+^ complex stability involved simultaneous data treatment obtained by UV-Vis (“batch” method samples prepared by varying their acid concentration, as well as by conventional UV-Vis titration performed in the pH range of 1.80–11.80) and those obtained by direct pH-potentiometric titration above pH = 1.75. The ligand concentration in the samples was 3.37 mM and the metal-to-ligand concentration ratio was 1:1 in the samples. During the fitting of the UV-Vis data molar absorption coefficients of Cu^2+^ and that of the final deprotonated complexes (Cu(DO3A-ACE)^2−^ and Cu(DO3A-BACE)^2−^) were fixed (based on the data determined in a separate experiment at 3–4 different concentrations), while those of the protonated species were refined simultaneously during the stability data refinement.

Direct pH-potentiometric titrations were performed in the pH range 1.78–11.80 with a Methrohm 888 Titrando titration workstation and a Metrohm-6.0233.100 combined electrode (Metrohm AG, Herisau, Switzerland). The stirred samples (6.00 mL) were thermostated at 25 °C, the ionic strength of the solutions was kept constant (0.15 M NaCl) and the titrations were performed under an inert gas atmosphere N_2._ KH-phtalate (Aldrich and Sigma, 99.95–100.05%, pH = 4.005) and borax (Aldrich and Sigma, 99.5%, pH = 9.177) were used to calibrate the pH meter. A 0.01 M HCl solution was titrated with standardized NaOH solution (Millipore-Sigma, St. Louis, MO, USA). The differences between the measured and calculated pH values were used to calculate the H^+^ concentrations from the experimentally measured pH values as described by Irving et al. [74]. The ion product of water was determined from the same HCl-NaOH titration experiments over the pH range of about 11.20–11.80 (pK_w_ = 13.845).

The determination of stabilities of Ce^3+^, Gd^3+^, and Yb^3+^ complexes was performed by the out-of-cell technique. Separate samples of 2.0 mL containing the ligand and the metal ion at 2.00 mM were prepared by setting their acid concentration so that the pH in the samples at equilibrium was expected to be in the pH range of 2–4 using model calculations. The samples were set aside for 12 weeks to attain the equilibrium before their pH was measured (the time required to reach the equilibrium was estimated based on UV-Vis measurements performed for Ce^3+^ complexes). The stability constants of the Ce^3+^ complexes were also determined using UV-Vis spectrophotometry at 312 nm (corresponding to 4f^1^ to 4f^0^5d^1^ transition), in 0.15 M NaCl aqueous solutions at 25 °C, whereas the stability constants of the Gd^3+^ complexes were also determined by water proton T_1_ relaxometry at 25 °C set by a circulation water bath. The complex formation in these samples was studied in mixtures containing 1.00 mM Ln^3+^ and 1.00 mM ligand.

The protonation (Equation (8)) and stability constants (Equations (9) and (10)) were calculated from the titration data using the program PSEQUAD (where I = 1, 2, and 3) [75]. The errors given correspond to one standard deviation.

(9) $$K_{\mathrm{ML}}=\frac{\;\left[\mathrm{ML}\right]}{\left[M\right]\left[L\right]\;}$$

and

(10) $$K_{ML}^{H_{i}}=\frac{\;\left[MH_{i}L\right]}{\left[MH_{i-1}L\right]\left[H^{+}\right]}$$

### 3.4. UV-Vis Spectrophotometry

Formation rates of the Eu^3+^ complexes were studied by spectrophotometry (JASCO V750 UV-Vis spectrophotometer; Waltham, MA, USA) at 255 nm in the presence of a 5- to 25-fold Eu^3+^ excess in order to assure the pseudo-first-order conditions. The pH range applied in the study was pH = 4.51–5.59, and the Eu^3+^ concentration was set to 1.6 mM. The pH in the samples was maintained constant by the application of 0.05 M N-methyl-piperazine (NMP) buffer (log *K*_1_^H^ = 4.78(3) determined under similar conditions. The pseudo-first-order rate constants (*k*_obs_) were calculated by fitting the absorbance values to Equation (11). The proton assisted dissociation of the Ce^3+^ complexes was investigated by UV-Vis measurements, where the complex concentration was 1.0 mM and the acid concentration was varied in the concentration range of 0.025–0.25 M (DO3A-ACE^4−^) and 0.1 to 1.0 M (DO3A-BACE^4−^). The proton assisted dissociation of the Gd^3+^ complexes (determined in the same H^+^-ion concentration as noted for the Ce^3+^ complexes above) and their exchange reactions with Zn^2+^ were studied by water proton *T*_1_ relaxometry (25 °C). The complex concentration in these experiments was 2.0 mM, while the Zn^2+^ concentration varied between 20.0 and 80.0 mM. In all studies, the ionic strength of the solutions was kept constant (0.15 M NaCl).

The pseudo-first-order rate constants (*k*_obs_) were calculated by fitting the relaxation rate data to Equation (11):

(11) $$X_{t}=\left(X_{0}-X_{e}\right)e^{\left(-k_{obs}t\right)}X_{e}$$

where *X*_0_, *X_e_*, and *X_t_* are the absorbance/relaxivity values at the start (*t* = 0), at equilibrium, and at the time *t*, respectively. The calculations were performed using the computer program Micromath Scientist, version 2.0 (Salt Lake City, UT, USA).

### 3.5. NMR Experiments

The longitudinal water proton relaxation times (*T*_1_) were measured using a Bruker Minispec MQ20 relaxometer operating at 0.49 T and a standard inversion-recovery sequence (180°-τ-90°) using 12–14 τ values (Bruker, Billerica, MA, USA). The sample relaxivities, *r*_1_, were calculated from the measured 1/*T*_1*obs*_ relaxation rates according to Equation (12), where 1/*T*_1w_ is the relaxation rate of water at the given temperature, and [Gd] is the Gd^3+^ concentration in mM.

(12) $$\frac{1}{T_{1obs}}=\frac{1}{T_{1w}}+r_{1}x\left[Gd\right]$$

A typical 90° pulse width was 3.24 μs, and the reproducibility of the *T_1_* data was 0.5–1.0%. The temperature was controlled with the use of circulating water bath (25 ± 0.1 °C).

^1^H NMR measurements were performed by a Bruker Avance II 400 spectrometer (9.4 T, 400 MHz) (Bruker, Billerica, MA, USA) equipped with a Bruker BCU II smart cooler using a 5-mm broad-band probe. The structural behavior and the dynamic processes of the Y(DO3A-ACE)^−^ and Y(DO3A-BACE)^−^ complexes were followed by 1D and 2D (COSY and NOESY) NMR spectroscopy.

The complexes were prepared in D_2_O ([Y(L)] = 0.02 M). The ^1^H chemical shifts are reported in ppm, with respect to TMS as an external standard (0 ppm). The COSY and NOESY spectra were collected using gradient pulses in the z direction with the standard Bruker pulse programs. For NOESY spectra, the mixing time (D8) was 1000 ms.

## 4. Conclusions

The DO3A-ACE^4−^ ligand has been synthesized and its complex formed with the Gd^3+^ ion characterized for several years in the search for the right bifunctional ligands (BFCs) capable of Ln^3+^ complexation [20,21,22,23,24]. Yet, studies aiming at characterization of the Ln^3+^ complexes from the thermodynamic and kinetic point of view (formation and dissociation of Ln^3+^ complexes) are lacking in the literature. In this work, the protonation constants of two DOTA derivative ligands, including the original DO3A-ACE^4−^ and the model amide derivative DO3A-BACE^4−^, and the stability and protonation constants of their complexes formed with some alkaline earth, transition metal, and lanthanide ions (representing large, medium, and small sized metal ions) are investigated by means of pH-potentiometric, UV-Vis spectrophotometric, and NMR methods at 25 °C in 0.15 M NaCl. The stability constants of the DO3A-ACE^4−^ and DO3A-BACE^4−^ complexes formed with Mg^2+^ and Ca^2+^ ions are lower, while the stability constants of the Zn^2+^ and Cu^2+^ complexes are similar and higher than those of the DOTA^4−^ and DO3A^3−^ complexes. The stability constants of the lanthanide(III) complexes of DO3A-BACE^4−^ increase from the beginning to the middle of the lanthanide series, then they remain practically constant for the heavier Ln^3+^ ions (represented by Yb^3+^). The stability constants of the Ln^3+^ complexes determined with different methods are in a good agreement with each other. The stability constants of the Ln(DO3A-BACE)^−^ complexes are several orders of magnitude lower than those of the DOTA^4−^ complex. A possible explanation of this phenomenon can be the presence of the amino-propionate pendant arm which is capable of forming a six membered chelate ring with the Ln^3+^ ions. On the other hand, the given pendant, along with the three acetate groups, can be an intramolecular competitor “pulling out”/”holding away” the metal ion from the cavity of the macrocycle.

The formation of the Eu^3+^ complex of the DO3A-ACE^4−^ ligand is significantly slower than that observed for Eu(DOTA)^−^, which is the exact opposite of the trend observed for the Ln^3+^ complexes of the DO3A-*N*prop^4−^ ligand. This difference can be explained by the appearance of the amine group in its protonated form under the conditions applied in the studies (as well as near the physiological pH), thereby lowering the stability constant (and the amount of protonated complex) of the intermediate, as well as its rate of conversion to the final complex (having the metal ion encapsulated). The same group is responsible for the “labilization” of Ln(DO3A-ACE)^−^ complexes, as evidenced by dissociation kinetic studies. We found that the kinetic inertness of the DO3A-ACE^4−^ complexes is higher than that of the DO3A^3−^ complexes, but dissociate several orders of magnitude faster than the complexes of the DOTA^4−^ ligand. The kinetic inertness of DO3A-BACE complexes are similar to that evidenced for the corresponding Ln(DOTA)^−^ complexes. To get more information of the decomplexation reactions of Gd(DO3A-ACE)^−^ complex, the exchange reactions occurring with Zn^2+^ have also been investigated as a function of pH at different exchanging metal concentrations. We found that the rate of the reactions decreases with the increasing concentration of the exchanging metal which indicates the formation of a “dead-end complex”, whereas the rate of acid assisted dissociation of the complex agrees relatively well with data determined by studying the acid assisted dissociation in fairly acidic samples.

The ^1^H NMR spectra of the diamagnetic Y(DO3A-ACE)^−^ and Y(DO3A-BACE)^−^ complexes, quite broad at low temperature, sharpen up at 80 °C, reflecting the dynamics of the intramolecular isomerization process between the SA pair of enantiomers, R-*Λ*(*λλλλ*) and S-*Δ*(*δδδδ*). The spectral assignments of the protons in the pendant arms, obtained on the basis of COSY spectra at 80 °C, allowed obtaining their vicinal coupling constants and relevant dihedral angles and conformations along the C_1ab_-C_1s_ bond of the C_α_-substituted pendant arm. Their comparison showed that the bulky substituent Ph(CO)NH is further away from the macrocyclic ring in Y(DO3A-BACE)^−^ than the amino group in Y(DO3A-ACE)^−^ to minimize the steric hindrance. This is supported by the lack of NOE cross peaks between the aromatic protons and either the ring or the arm protons in the 2D NOESY spectrum of Y(DO3A-BACE)^−^. The NOESY spectra of the two complexes show cross peaks between the arms and ring protons and between ring protons which are not present in the COSY spectra, indicating spatial proximities which reflect compact ligand structures in both complexes. The temperature dependence of the ^1^H 1D and NOESY spectra show that the internal ring motions are slower than the pendant arms rearrangements in both complexes, although ring dynamics are faster in Y(DO3A-BACE)^−^ than in Y(DO3A-ACE)^−^, probably due to the different conformations of the bulky arm substituent in the first complex which minimizes steric hindrance.

In summary, despite the significantly lower stability of Gd(DO3A-BACE)^−^ relative to Gd(DOTA)^−^, its similar kinetic inertness relative to acid assisted and Zn^2+^ mediated dissociation is similar to Gd(DOTA)^−^. This indicates the usefulness of the bifunctional DO3A-ACE in the design of GBCAs and Ln^3+^-based tags for protein structural NMR.

## Figures and Tables

**Table 1:** Protonation constants of DO3A-ACE^4−^, DO3A-BACE^4−^, DOTA^4−^, DO3A^3−^, and DO3A-*N*prop^4−^ at 25 °C.

	DO3A-ACE^4^^− a^	DO3A-BACE^4^^− a^	DOTA^4^^−^	DO3A-*N*prop^4^^− i^	DO3A-AE^3− j^	DO3A^3^^− k^
log *K*_1_^H^	9.98 (2)	9.40 (2)	12.6 ^c^; 11.22 ^d^; 12.09 ^e^; 11.09 ^f^; 11.36 ^g^; 9.37 ^h^	11.00	12.49	11.59
log *K*_2_^H^	9.54 (1)	8.57 (2)	9.70 ^c^; 9.64 ^d^; 9.76 ^e^; 9.23 ^f^; 9.73 ^g^; 9.14 ^h^	9.34	10.38	9.24
log *K*_3_^H^	8.58 (2)	4.39 (3)	4.50 ^c^; 4.86 ^d^; 4.56 ^e^; 4.24 ^f^; 4.54 ^g^; 4.63 ^h^	4.64	8.80	4.43
log *K*_4_^H^	4.10 (3)	3.29 (3)	4.14 ^c^; 3.68 ^d^; 4.09 ^e^; 4.18 ^f^; 4.41 ^g^; 3.91 ^h^	4.00	4.03	3.48
log *K*_5_^H^	2.16 (3)	2.33 (3)	2.32 ^c^; - ^d^; - ^e^; 1.88 ^f^; - ^g^; 1.99 ^h^	2.67	1.70	-
log *K*_6_^H^	-	-	-^c,d,e^ 1.71 ^f^, - ^g,h^	1.6	-	-
Σ log *K*_i_^H, b^	24.38	25.65	30.94 ^c^; 29.40 ^d^; 30.50 ^e^; 28.74 ^f^; 29.74 ^g^; 26.86 ^h^	28.98	28.60	28.65

^a^ I = 0.15 M NaCl, this work; ^b^ i = 1–4, the first four values involving macrocyclic N and carboxylate O atoms; ^c^ Ref. [26], I = 0.1 Me_4_NCl; ^d^ Ref. [27], I = 0.1 M KCl; ^e^ Ref. [28], I = 0.1 M Me_4_NNO_3_; ^f^ Ref. [29], I = 1.0 M NaCl (corrected); ^g^ Ref. [30], I = 0.1 M KCl; ^h^ Ref. [31], I = 0.1 M NaCl; ^i^ Ref. [19], I = 0.1 M KCl; ^j^ Ref. [32], I = 0.1 M KCl; ^k^ Ref. [33], I = 0.1 M Me_4_NCl.

**Table 4:** Rate constants k_OH_ (M^−1^s^−1^) characterizing the formation of Eu^3+^ and/or Gd^3+^ macrocyclic complexes (25 °C).

Ligand	Eu^3+^ Complexes	Gd^3+^ Complexes
DO3A-ACE^4−^	1.10(3) × 10^6 a^	-
DOTA^4−^	1.1 × 10^7 b^	5.9 × 10^6 c^
DO3A-butriol^3−^	4.8 × 10^6 d^	-
DO3A-*N*prop^4−^	-	2.9 × 10^7 e^
DO3A-pic^4−^	3.7 × 10^7 f^	-
DO3A-AE^3−^	1.7 × 10^6 g^	
DO3A-DMAE^3−^	1.8 × 10^6 g^	

^a^ This work; ^b^ Ref. [45]; ^c^ Ref. [48]; ^d^ Ref. [49]; ^e^ Ref. [19]; ^f^ Ref. [50]; ^g^ Ref. [32].

**Table 5:** Comparison of the rate constants of spontaneous and acid catalyzed dissociation for various Ce^3+^ and Gd^3+^ complexes, and for the exchange reaction of Gd(DO3A-ACE)^−^ with Zn^2+^ (25 °C).

Ligand	Ln^3+^	*k*_0_ (s^−1^)	*k*_1_ (M^−1^s^−1^)	*k*_2_ (M^−2^s^−1^)
DO3A-ACE^4− a^	Ce	-	(8.4 ± 0.1) × 10^−2^	-
	Gd	-	(6.9 ± 0.1) × 10^−3^	-
Zn^2+^ exchange ^b^	Gd	(7 ± 4) × 10^−8^	(8.7 ± 0.9) × 10^−3^	-
DO3A-BACE^4− a^	Ce	(6.6 ± 6.0) × 10^−5^	(1.9 ± 0.2) × 10^−3^	(1.2 ± 0.2) × 10^−3^
	Gd	(2.8 ± 3.1) × 10^−6^	(9.3 ± 1.3) × 10^−5^	(1.5 ± 1.2) × 10^−5^
DOTA^4− c^	Ce	-	8 × 10^−4^	2.0 × 10^−3^
	Gd	5 × 10^−10^	2.0 × 10^−5^	-
DO3A^3− d^	Ce	1.8 × 10^−3^	1.12 × 10^−1^	-
	Gd	4.4 × 10^−4^	2.51 × 10^−2^	-
DO3A-*N*prop^4− e^	Ce	-	7 × 10^−4^	0.51

^a^ This work; ^b^ the protonation constants as well as the stability were also determined as follows: *K*_H_ = (3.7 ± 1.5)·10^3^ M^−1^ and *K*_M_ = (53 ± 22) M^−1^; ^c^ Refs. [41,45]; ^d^ Ref. [41]; ^e^ Ref. [19].

## Data Availability

All Supplementary Materials can be found at MDPI. Our data will also be made available to any investigator upon request.

## Supplementary Materials

The following are available online: Additional figures displaying equilibrium (pH-potentiometric titration curves of M(II) complexes formed with DO3A-BACE), spectral (absorption spectra of the Cu(DO3A-BACE)^2−^ as a function of pH, changes in the absorption spectra observed during the formation of Ce(III) and Eu(DO3A-ACE)-complex, ^1^H-NMR spectra and their temperature dependence observed for the Y(III) complexes of DO3A-ACE and BACE ligands, as well as the 2D NOESY spectra recorded at 80 °C), and kinetic data (dependence of pseudo-first-order rate constants (*k*_obs_) on pH and Zn(II) ion concentration for the exchange reaction of Gd(DO3A-ACE)-occurring with Zn(II)). For Supplementary Materials and crystallographic data in CIF or other electronic format.

## Sample Availability

Samples of the compounds are available from the authors.

## References

[1] Caravan P., Ellison J.J., McMurry T.J., Lauffer R.B. (1999). Gadolinium(III) Chelates as MRI Contrast Agents: Structure, Dynamics, and Applications. Chem. Rev..

[2] Helm L., Merbach A.E., Tóth É. (2013). The Chemistry of Contrast Agents in Medical Magnetic Resonance Imaging.

[3] Geraldes C.F.G.C., Laurent S. (2009). Classification and Basic Properties of Contrast Agents for Magnetic Resonance Imaging. Contrast Media Mol. Imaging.

[4] Grobner T. (2006). Gadolinium—A Specific Trigger for the Development of Nephrogenic Fibrosing Dermopathy and Nephrogenic Systemic Fibrosis?. Nephrol. Dial. Transplant..

[5] Marckmann P., Skov L., Rossen K., Dupont A., Damholt M.B., Heaf J.G., Thomsen H.S. (2006). Nephrogenic Systemic Fibrosis: Suspected Causative Role of Gadodiamide Used for Contrast-Enhanced Magnetic Resonance Imaging. J. Am. Soc. Nephrol..

[6] Birka M., Wentker K.S., Lusmöller E., Arheilger B., Wehe C.A., Sperling M., Stadler R., Karst U. (2015). Diagnosis of Nephrogenic Systemic Fibrosis by Means of Elemental Bioimaging and Speciation Analysis. Anal. Chem..

[7] Kanda T., Ishii K., Kawaguchi H., Kitajima K., Takenaka D. (2014). High Signal Intensity in the Dentate Nucleus and Globus Pallidus on Unenhanced *T*_1_-Weighted MR Images: Relationship with Increasing Cumulative Dose of a Gadolinium-Based Contrast Material. Radiology.

[8] Kanal E., Tweedle M.F. (2015). Residual or Retained Gadolinium: Practical Implications for Radiologists and Our Patients. Radiology.

[9] McDonald R.J., McDonald J.S., Kallmes D.F., Jentoft M.E., Murray D.L., Thielen K.R., Williamson E.E., Eckel L.J. (2015). Intracranial Gadolinium Deposition after Contrast-Enhanced MR Imaging. Radiology.

[10] Port M., Idée J.-M., Medina C., Robic C., Sabatou M., Corot C. (2008). Efficiency, Thermodynamic and Kinetic Stability of Marketed Gadolinium Chelates and Their Possible Clinical Consequences: A Critical Review. Biometals.

[11] Tircsó G., Molnár E., Csupász T., Garda Z., Botár R., Kálmán F.K., Kovács Z., Brücher E., Tóth I., Sigel A., Freisinger E., Sigel R.K.O. (2021). Metal Ions in Bio-Imaging Techniques.

[12] Wahsner J., Gale E.M., Rodríguez-Rodríguez A., Caravan P. (2019). Chemistry of MRI Contrast Agents: Current Challenges and New Frontiers. Chem. Rev..

[13] Caravan P. (2006). Strategies for Increasing the Sensitivity of Gadolinium Based MRI Contrast Agents. Chem. Soc. Rev..

[14] Ruloff R., Tóth É., Scopelliti R., Tripier R., Handel H., Merbach A.E. (2002). Accelerating Water Exchange for Gd^III^ Chelates by Steric Compression around the Water Binding Site. Chem. Commun..

[15] Laus S., Ruloff R., Tóth É., Merbach A.E. (2003). Gd^III^ Complexes with Fast Water Exchange and High Thermodynamic Stability: Potential Building Blocks for High-Relaxivity MRI Contrast Agents. Chem. Eur. J..

[16] Torres S., Martins J.A., André J.P., Geraldes C.F.G.C., Merbach A.E., Tóth É. (2006). Supramolecular Assembly of an Amphiphilic Gd^III^ Chelate: Tuning the Reorientational Correlation Time and the Water Exchange Rate. Chem. Eur. J..

[17] Jászberényi Z., Sour A., Tóth É., Benmelouka M., Merbach A.E. (2005). Fine-Tuning Water Exchange on Gd^III^ Poly(Amino Carboxylates) by Modulation of Steric Crowding. Dalton Trans..

[18] Balogh E., Tripier R., Ruloff R., Tóth É. (2005). Kinetics of Formation and Dissociation of Lanthanide(iii) Complexes with the 13-Membered Macrocyclic Ligand TRITA^4−^. Dalton Trans..

[19] Balogh E., Tripier R., Fousková P., Reviriego F., Handel H., Tóth É. (2007). Monopropionate Analogues of DOTA^4–^ and DTPA^5–^: Kinetics of Formation and Dissociation of Their Lanthanide(III) Complexes. Dalton Trans..

[20] Ferreira M.F., Martins A.F., Martins J.A., Ferreira P.M., Tóth É., Geraldes C.F.G.C. (2009). Gd(DO3A-N-α-Aminopropionate): A Versatile and Easily Available Synthon with Optimized Water Exchange for the Synthesis of High Relaxivity, Targeted MRI Contrast Agents. Chem. Commun..

[21] Ferreira M.F., Mousavi B., Ferreira P.M., Martins C.I.O., Helm L., Martins J.A., Geraldes C.F.G.C. (2012). Gold Nanoparticles Functionalised with Stable, Fast Water Exchanging Gd^3+^ Chelates as High Relaxivity Contrast Agents for MRI. Dalton Trans..

[22] Ferreira M.F., Martins A.F., Martins C.I.O., Ferreira P.M., Tóth É., Rodrigues T.B., Calle D., Cerdan S., López-Larrubia P., Martins J.A. (2013). Amide Conjugates of the DO3A- *N* -(*α* -Amino)Propionate Ligand: Leads for Stable, High Relaxivity Contrast Agents for MRI?: AMIDE CONJUGATES OF THE DO3A- *N* -(*α* -AMINO)PROPIONATE LIGAND. Contrast Media Mol. Imaging.

[23] Ferreira M.F., Pereira G., Martins A.F., Martins C.I.O., Prata M.I.M., Petoud S., Toth E., Ferreira P.M.T., Martins J.A., Geraldes C.F.G.C. (2014). Ln[DO3A-N-α-(Pyrenebutanamido)Propionate] Complexes: Optimized Relaxivity and NIR Optical Properties. Dalton Trans..

[24] Ferreira M.F., Gonçalves J., Mousavi B., Prata M.I.M., Rodrigues S.P.J., Calle D., López-Larrubia P., Cerdan S., Rodrigues T.B., Ferreira P.M. (2015). Gold Nanoparticles Functionalised with Fast Water Exchanging Gd^3+^ Chelates: Linker Effects on the Relaxivity. Dalton Trans..

[25] Boros E., Polasek M., Zhang Z., Caravan P. (2012). Gd(DOTAla): A Single Amino Acid Gd-Complex as a Modular Tool for High Relaxivity MR Contrast Agent Development. J. Am. Chem. Soc..

[26] Burai L., Fábián I., Király R., Szilágyi E., Brücher E. (1998). Equilibrium and Kinetic Studies on the Formation of the Lanthanide(III) Complexes, [Ce(Dota)]− and [Yb(Dota)]− (H_4_dota = 1,4,7,10-Tetraazacyclododecane-1,4,7,10-Tetraacetic Acid). J. Chem. Soc. Dalton Trans..

[27] Clarke E.T., Martell A.E. (1991). Stabilities of the Alkaline Earth and Divalent Transition Metal Complexes of the Tetraazamacrocyclic Tetraacetic Acid Ligands. Inorg. Chim. Acta.

[28] Chaves S., Delgado R., Da Silva J.J.R.F. (1992). The Stability of the Metal Complexes of Cyclic Tetra-Aza Tetra-Acetic Acids. Talanta.

[29] Desreux J.F., Merciny E., Loncin M.F. (1981). Nuclear Magnetic Resonance and Potentiometric Studies of the Protonation Scheme of Two Tetraaza Tetraacetic Macrocycles. Inorg. Chem..

[30] Stetter H., Frank W. (1976). Complex Formation with Tetraazacycloalkane-*N,N′,N″,N‴*-Tetraacetic Acids as a Function of Ring Size. Angew. Chem. Int. Ed..

[31] Kumar K., Chang C.A., Francesconi L.C., Dischino D.D., Malley M.F., Gougoutas J.Z., Tweedle M.F. (1994). Synthesis, Stability, and Structure of Gadolinium(III) and Yttrium(III) Macrocyclic Poly(Amino Carboxylates). Inorg. Chem..

[32] Baranyai Z., Rolla G.A., Negri R., Forgács A., Giovenzana G.B., Tei L. (2014). Comprehensive Evaluation of the Physicochemical Properties of Ln^III^ Complexes of Aminoethyl-DO3A as pH-Responsive *T*_1_-MRI Contrast Agents. Chem. Eur. J..

[33] Kumar K., Tweedle M.F., Malley M.F., Gougoutas J.Z. (1995). Synthesis, Stability, and Crystal Structure Studies of Some Ca^2+^, Cu^2+^, and Zn^2+^ Complexes of Macrocyclic Polyamino Carboxylates. Inorg. Chem..

[34] Delgado R., da Silva J.J.R.F. (1982). Metal Complexes of Cyclic Tetra-Azatetra-Acetic Acids. Talanta.

[35] Chang C.A. (1996). Selectivity of Macrocyclic Aminocarboxylates for Alkaline-Earth Metal Ions and Stability of Their Complexes. J. Chem. Soc. Dalton Trans..

[36] Takács A., Napolitano R., Purgel M., Bényei A.C., Zékány L., Brücher E., Tóth I., Baranyai Z., Aime S. (2014). Solution Structures, Stabilities, Kinetics, and Dynamics of DO3A and DO3A–Sulphonamide Complexes. Inorg. Chem..

[37] Riesen A., Zehnder M., Kaden T.A. (1986). Metal complexes of macrocyclic ligands. Part XXIII. Synthesis, properties, and structures of mononuclear complexes with 12- and 14-membered tetraazamacrocycle-*N,N′,N″,N‴*-tetraacetic Acids. Helv. Chim. Acta.

[38] Cacheris W.P., Nickle S.K., Sherry A.D. (1987). Thermodynamic Study of Lanthanide Complexes of 1,4,7-Triazacyclononane-*N,N’,N”*-Triacetic Acid and 1,4,7,10-Tetraazacyclododecane-*N,N′,N″,N‴*-Tetraacetic Acid. Inorg. Chem..

[39] Tóth Ė., Brücher E. (1994). Stability Constants of the Lanthanide(III)-1,4,7,10-Tetraazacyclododecane-*N,N′,N″,N‴*-Tetraacetate Complexes. Inorg. Chim. Acta.

[40] Wang X., Jin T., Comblin V., Lopez-Mut A., Merciny E., Desreux J.F. (1992). A Kinetic Investigation of the Lanthanide DOTA Chelates. Stability and Rates of Formation and of Dissociation of a Macrocyclic Gadolinium(III) Polyaza Polycarboxylic MRI Contrast Agent. Inorg. Chem..

[41] Kumar K., Chang C.A., Tweedle M.F. (1993). Equilibrium and Kinetic Studies of Lanthanide Complexes of Macrocyclic Polyamino Carboxylates. Inorg. Chem..

[42] Gündüz S., Vibhute S., Botár R., Kálmán F.K., Tóth I., Tircsó G., Regueiro-Figueroa M., Esteban-Gómez D., Platas-Iglesias C., Angelovski G. (2018). Coordination Properties of GdDO3A-Based Model Compounds of Bioresponsive MRI Contrast Agents. Inorg. Chem..

[43] Gritmon T.F., Goedken M.P., Choppin G.R. (1977). The Complexation of Lanthanides by Aminocarboxylate Ligands—I: Stability Constants. J. Inorg. Nucl. Chem..

[44] Tircsó G., Kovács Z., Sherry A.D. (2006). Equilibrium and Formation/Dissociation Kinetics of Some Ln^III^ PCTA Complexes. Inorg. Chem..

[45] Toth E., Brucher E., Lazar I., Toth I. (1994). Kinetics of Formation and Dissociation of Lanthanide(III)-DOTA Complexes. Inorg. Chem..

[46] Chang C.A., Chen Y.-H., Chen H.-Y., Shieh F.-K. (1998). Capillary Electrophoresis, Potentiometric and Laser Excited Luminescence Studies of Lanthanide(III) Complexes of 1,7-Dicarboxymethyl-1,4,7,10-Tetraazacyclododecane (DO2A). J. Chem. Soc. Dalton Trans..

[47] Tircsó G., Tircsóné Benyó E., Garda Z., Singh J., Trokowski R., Brücher E., Sherry A.D., Tóth É., Kovács Z. (2020). Comparison of the Equilibrium, Kinetic and Water Exchange Properties of Some Metal Ion-DOTA and DOTA-bis(Amide) Complexes. J. Inorg. Biochem..

[48] Kumar K., Tweedle M.F. (1993). Ligand Basicity and Rigidity Control Formation of Macrocyclic Polyamino Carboxylate Complexes of Gadolinium(III). Inorg. Chem..

[49] Szilágyi E., Tóth É., Kovács Z., Platzek J., Radüchel B., Brücher E. (2000). Equilibria and Formation Kinetics of Some Cyclen Derivative Complexes of Lanthanides. Inorg. Chim. Acta.

[50] Regueiro-Figueroa M., Bensenane B., Ruscsák E., Esteban-Gómez D., Charbonnière L.J., Tircsó G., Tóth I., de Blas A., Rodríguez-Blas T., Platas-Iglesias C. (2011). Lanthanide Dota-like Complexes Containing a Picolinate Pendant: Structural Entry for the Design of Ln ^III^ -Based Luminescent Probes. Inorg. Chem..

[51] Táborský P., Svobodová I., Lubal P., Hnatejko Z., Lis S., Hermann P. (2007). Formation and Dissociation Kinetics of Eu(III) Complexes with H5do3ap and Similar Dota-like Ligands. Polyhedron.

[52] Táborský P., Lubal P., Havel J., Kotek J., Hermann P., Lukeš I. (2005). Thermodynamic and Kinetic Studies of Lanthanide(III) Complexes with H5do3ap (1,4,7,10-Tetraazacyclododecane-1,4,7-Triacetic-10-(Methylphosphonic Acid)), a Monophosphonate Analogue of H4dota. Collect. Czech. Chem. Commun..

[53] Baranyai Z., Brücher E., Iványi T., Király R., Lázár I., Zékány L. (2005). Complexation Properties of *N*,*N′*,*N″*,*N**‴*-[1,4,7,10-Tetraazacyclododecane-1,4,7,10-Tetrayltetrakis(1-Oxoethane-2,1-Diyl)]Tetrakis[Glycine] (H_4_Dotagl). Equilibrium, Kinetic, and Relaxation Behavior of the Lanthanide(III) Complexes. Helv. Chim. Acta.

[54] Tóth É., Király R., Platzek J., Radüchel B., Brücher E. (1996). Equilibrium and Kinetic Studies on Complexes of 10-[2,3-Dihydroxy-(1-Hydroxymethyl)-Propyl]-1,4,7,10-Tetraazacyclododecane-1,4,7-Triacetate. Inorg. Chim. Acta.

[55] Baranyai Z., Pálinkás Z., Uggeri F., Maiocchi A., Aime S., Brücher E. (2012). Dissociation Kinetics of Open-Chain and Macrocyclic Gadolinium(III)-Aminopolycarboxylate Complexes Related to Magnetic Resonance Imaging: Catalytic Effect of Endogenous Ligands. Chem. Eur. J..

[56] Brücher E., Laurenczy G., Makra Z.S. (1987). Studies on the Kinetics of Formation and Dissociation of the Cerium(III)-DOTA Complex. Inorg. Chim. Acta.

[57] Sarka L., Burai L., Brücher E. (2000). The Rates of the Exchange Reactions between [Gd(DTPA)]^2−^ and the Endogenous Ions Cu^2+^ and Zn^2+^: A Kinetic Model for the Prediction of the In Vivo Stability of [Gd(DTPA)]^2−^, Used as a Contrast Agent in Magnetic Resonance Imaging. Chem. Eur. J..

[58] Peters J.A., Djanashvili K., Geraldes C.F.G.C., Platas-Iglesias C. (2020). The Chemical Consequences of the Gradual Decrease of the Ionic Radius along the Ln-Series. Coord. Chem. Rev..

[59] Aime S., Botta M., Ermondi G. (1992). NMR Study of Solution Structures and Dynamics of Lanthanide(III) Complexes of DOTA. Inorg. Chem..

[60] Aime S., Botta M., Fasano M., Marques M.P.M., Geraldes C.F.G.C., Pubanz D., Merbach A.E. (1997). Conformational and Coordination Equilibria on DOTA Complexes of Lanthanide Metal Ions in Aqueous Solution Studied by ^1^H-NMR Spectroscopy. Inorg. Chem..

[61] Hoeft S., Roth K. (1993). Struktur und Dynamik von Lanthanoid-Tetraazacyclododecantetraacetat-(DOTA-)Komplexen in Lösung. Chem. Ber..

[62] Jacques V., Desreux J.F. (1994). Quantitative Two-Dimensional EXSY Spectroscopy and Dynamic Behavior of a Paramagnetic Lanthanide Macrocyclic Chelate: YbDOTA (DOTA = 1,4,7,10-Tetraazacyclododecane-*N,N′,N″,N‴*-Tetraacetic Acid). Inorg. Chem..

[63] Woods M., Aime S., Botta M., Howard J.A.K., Moloney J.M., Navet M., Parker D., Port M., Rousseaux O. (2000). Correlation of Water Exchange Rate with Isomeric Composition in Diastereoisomeric Gadolinium Complexes of Tetra(Carboxyethyl)Dota and Related Macrocyclic Ligands. J. Am. Chem. Soc..

[64] Aime S., Botta M., Ermondi G., Terreno E., Anelli P.L., Fedeli F., Uggeri F. (1996). Relaxometric, Structural, and Dynamic NMR Studies of DOTA-like Ln(III) Complexes (Ln = La, Gd, Ho, Yb) Containing a *p* -Nitrophenyl Substituent. Inorg. Chem..

[65] André J.P., Brücher E., Kiraly R., Carvalho R.A., Mäcke H., Geraldes C.F.G.C. (2005). H5dotasa (=(ARS)-α-(Carboxymethyl)-1,4,7,10-Tetraazacyclododecane-1,4,7,10-Tetraacetic Acid), an Asymmetrical Derivative of H4dota (=1,4,7,10-Tetraazacyclododecane-1,4,7,10-Tetraacetic Acid) Substituted at One Acetate Pendant Arm: ^1^H-NMR and Potentiometri. Helv. Chim. Acta.

[66] Shukla R.B. (1995). Rotating-Frame Exchange Spectroscopy of Macrocyclic Systems. J. Magn. Reson. Ser. A.

[67] Brittain H.G., Desreux J.F. (1984). Luminescence and NMR Studies of the Conformational Isomers of Lanthanide Complexes with an Optically Active Polyaza Polycarboxylic Macrocycle. Inorg. Chem..

[68] Di Bari L., Pintacuda G., Salvadori P. (2000). Solution Equilibria in YbDOTMA, a Chiral Analogue of One of the Most Successful Contrast Agents for MRI, GdDOTA. Eur. J. Inorg. Chem..

[69] Ranganathan R.S., Raju N., Fan H., Zhang X., Tweedle M.F., Desreux J.F., Jacques V. (2002). Polymethylated DOTA Ligands. 2. Synthesis of Rigidified Lanthanide Chelates and Studies on the Effect of Alkyl Substitution on Conformational Mobility and Relaxivity. Inorg. Chem..

[70] Woods M., Kovacs Z., Kiraly R., Brücher E., Zhang S., Sherry A.D. (2004). Solution Dynamics and Stability of Lanthanide(III) (*S*)—2-(*p*-Nitrobenzyl)DOTA Complexes. Inorg. Chem..

[71] Karplus M. (1959). Contact Electron-Spin Coupling of Nuclear Magnetic Moments. J. Chem. Phys..

[72] Bodor A., Bányai I., Tóth I. (2002). 1H- and 13C-NMR as Tools to Study Aluminium Coordination Chemistry—Aqueous Al(III)–Citrate Complexes. Coord. Chem. Rev..

[73] Levitt M.H., Sheldrick C.M. (2001). Spin Dynamics.

[74] Irving H.M., Miles M.G., Pettit L.D. (1967). A Study of Some Problems in Determining the Stoicheiometric Proton Dissociation Constants of Complexes by Potentiometric Titrations Using a Glass Electrode. Anal. Chim. Acta.

[75] Zékány L., Nagypál I., Legett D.J. (1985). Computational Methods for Determination of Formation Constants.

