# Supplementary material for: Complexes of Bifunctional DO3A-N-(α-amino)propinate Ligands with Mg(II), Ca(II), Cu(II), Zn(II), and Lanthanide(III) Ions: Thermodynamic Stability, Formation and Dissociation Kinetics, and Solution Dynamic NMR Studies
Source: Molecules. 2021 Aug 16;26(16):4956. doi: 10.3390/molecules26164956 (PMC8398831; doi:10.3390/molecules26164956)
Supplement: Supplementary file 1 [file molecules-26-04956-s001.zip › molecules-1274293-supplementary.pdf]

Supplementary Material

Complexes of bifunctional DO3A-N-(α-amino)propinate ligands with Mg(II), Ca(II), Cu(II), Zn(II) and Lanthanide(III) ions: thermodynamic stability, formation and dissociation kinetics and solution NMR studies

Zoltán Garda[1], Tamara Kócs[1], István Bányai[1], José A. Martins[2], Ferenc K. Kálmán[1], Imre Tóth[1], Carlos F.G.C. Geraldes[3,4,5*] and Gyula Tircsó[1*]

[1]Department of Physical Chemistry, Faculty of Science and Technology, University of Debrecen, H-4010, Debrecen, Egyetem tér 1., Hungary.

[2]Centro de Química, Campus de Gualtar, Universidade do Minho, 4710-057 Braga, Portugal.

[3]Department of Life Sciences and [4]Coimbra Chemistry Centre, Faculty of Science and Technology, University of Coimbra, 3000-393 Coimbra, Portugal.

[5] IBIT/ICNAS - Instituto de Ciências Nucleares Aplicadas à Saúde. Pólo das Ciências da Saúde, Azinhaga de Santa Comba, 3000-548 Coimbra, Portugal.

Corresponding authors email: geraldes@uc.pt or gyula.tircso@science.unideb.hu

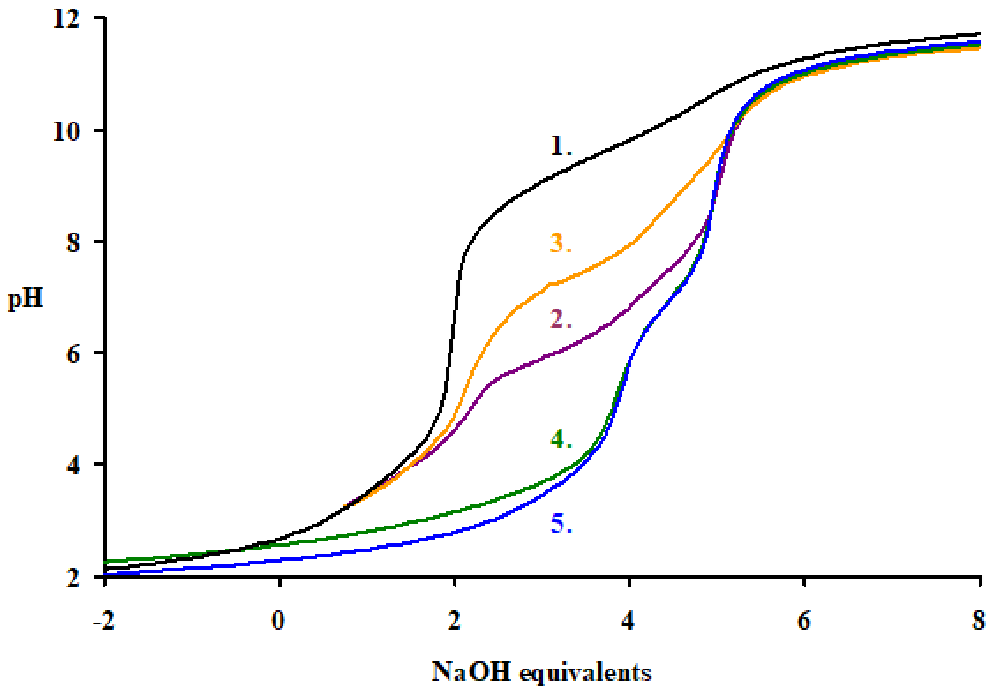

Figure S1. pH-potentiometric titration curve of the DO3A-ACE^{4-} ligand (1) at 3.29 mM concentration and samples containing 1:1 equivalents of Ca^{2+} (2), Mg^{2+} (3), Zn^{2+} (4) or Cu^{2+} (5) ions and the (25 °C, I = 0.15 M NaCl).

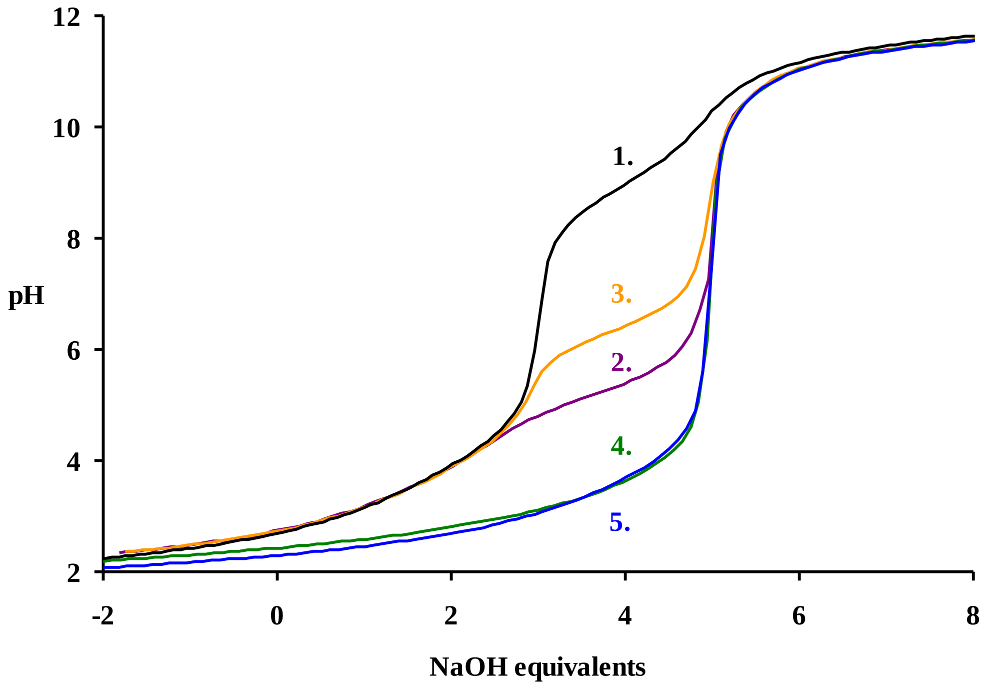

Figure S2. pH-potentiometric titration curve of the DO3A-BACE^{4-} ligand (1) at 2.38 mM concentration and samples containing 1:1 equivalents of Ca^{2+} (2), Mg^{2+} (3), Zn^{2+} (4) or Cu^{2+} (5) ions and the (25 °C, I = 0.15 M NaCl).

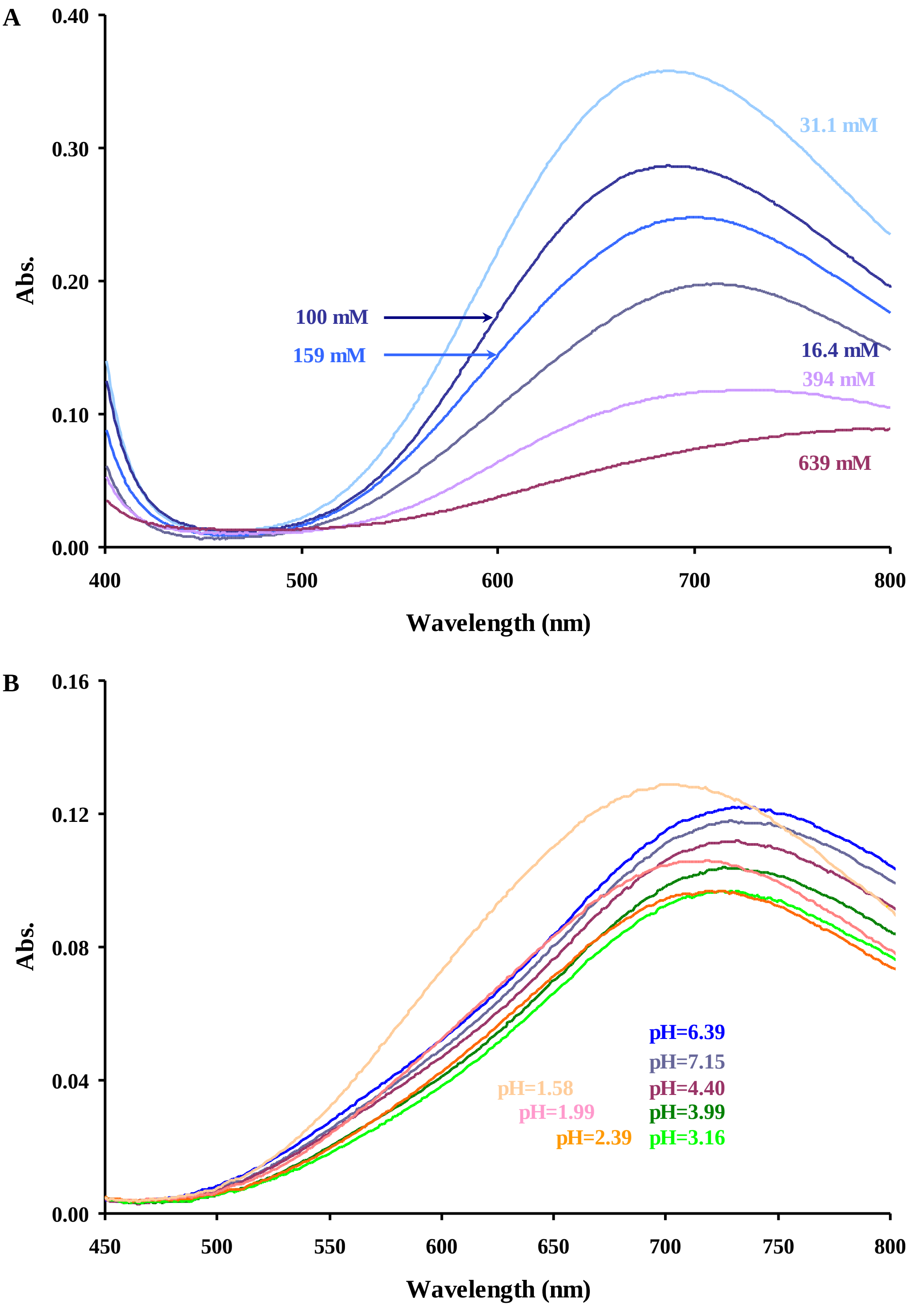

Figure S3. Absorption spectra of the Cu^{2+}–DO3A-$BACE^{4-}$–H^{+} system ([HCl]+[NaCl]=1.0 M; [Cu^{2+}]=[DO3A-BACE]=2.22 mM, l=1 cm, 25 °C). Top figure shows the batch samples prepared in the acid concentration range of 16.4 to 639 mM; Lower figure shows the spectra obtained in the pH range of pH=1.58 to 7.15.

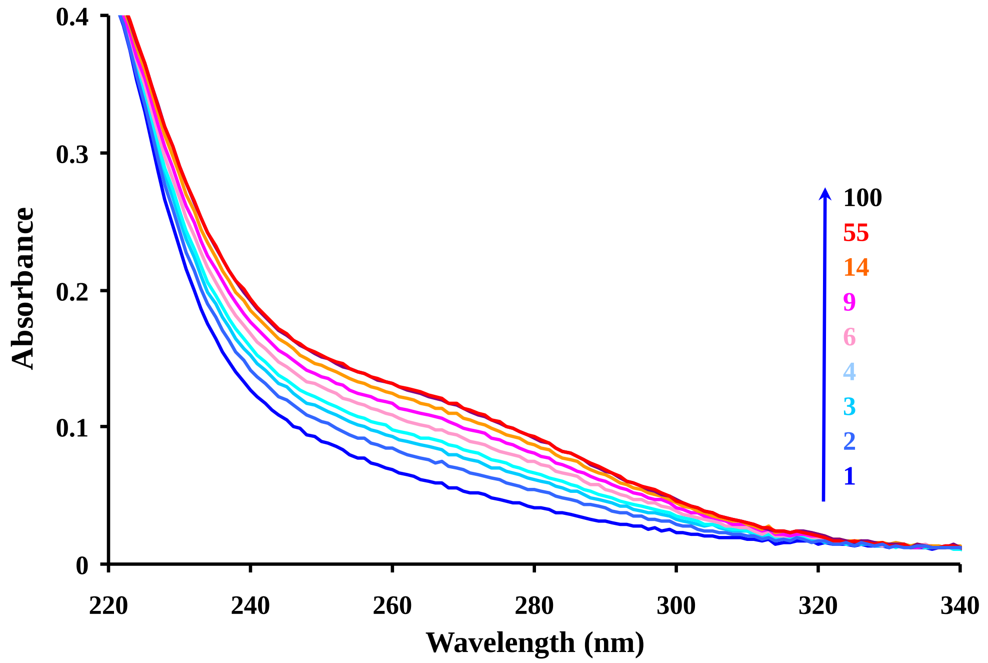

Figure S4. UV-Vis spectra recorded at different times t after mixing Eu^{3+} and DO3A-ACE at pH = 5.30 (0.05 M N-methylpiperazine; c_{Eu3+} = 2 × cDO3A-ACE = 4×10^{-4} M; 0.15 M NaCl, 25 °C). t = 1, 2, 3, 4, 6, 9, 14, 55 and 100 minutes after mixing (upward direction)

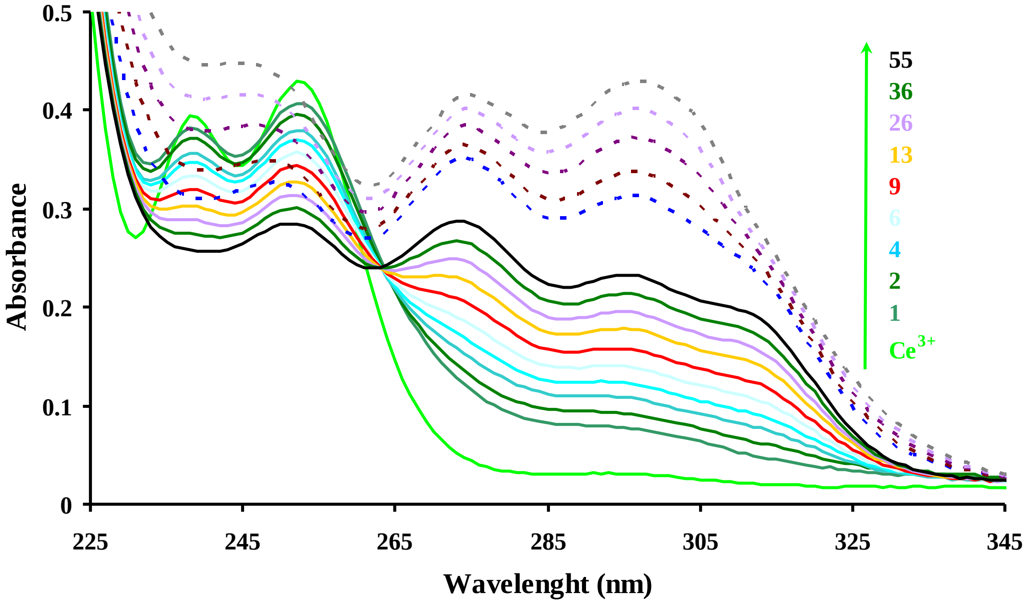

Figure S5. UV-Vis spectra recorded at different times t after mixing Ce^{3+} and DO3A-ACE at pH = 4.12 (0.05 M N,N'-dimethylpiperazine; c_{Ce} = 2x$c_{DO3A\text{-}ACE}$ = 4×10^{-4} M; 0.15 M NaCl, 25 °C). t = 1, 2, 4, 6, 9, 13, 26, 36, and 55 minutes after mixing (upward direction). The spectra plotted with dotted lines were taken after 5 min heating at 80 °C (exposed to air) and the changes indicates slow oxidation of Ce^{3+} to Ce^{4+}.

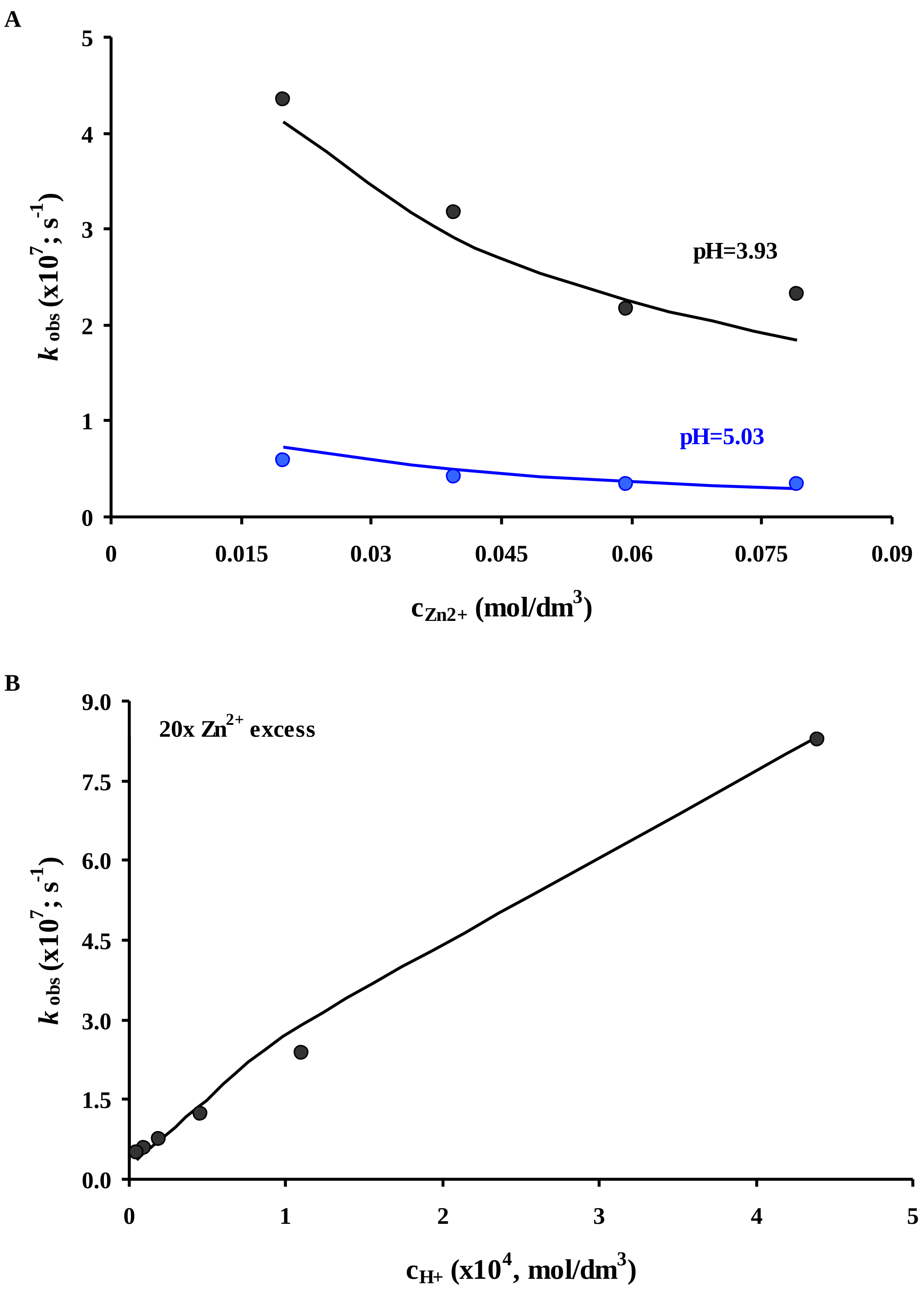

Figure S6. Dependence of pseudo-first-order rate constants (k_{obs}) A). on [Zn^{2+}] and B). on [H^+] concentration at [Zn^{2+}] = 40 mM for the exchange reaction of Gd(DO3A-ACE)$^-$ with Zn^{2+} at pH = 3.93 (♦) and pH = 5.03 (♦) (25 ºC).

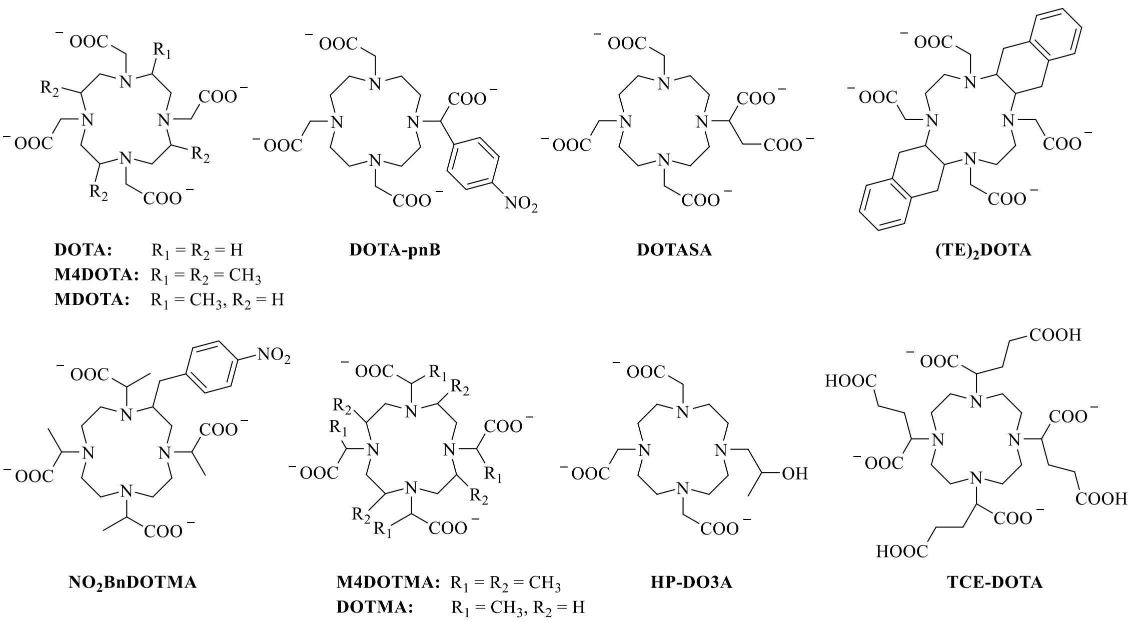

Figure S7. Formulae of the ligands mentioned in the NMR part.

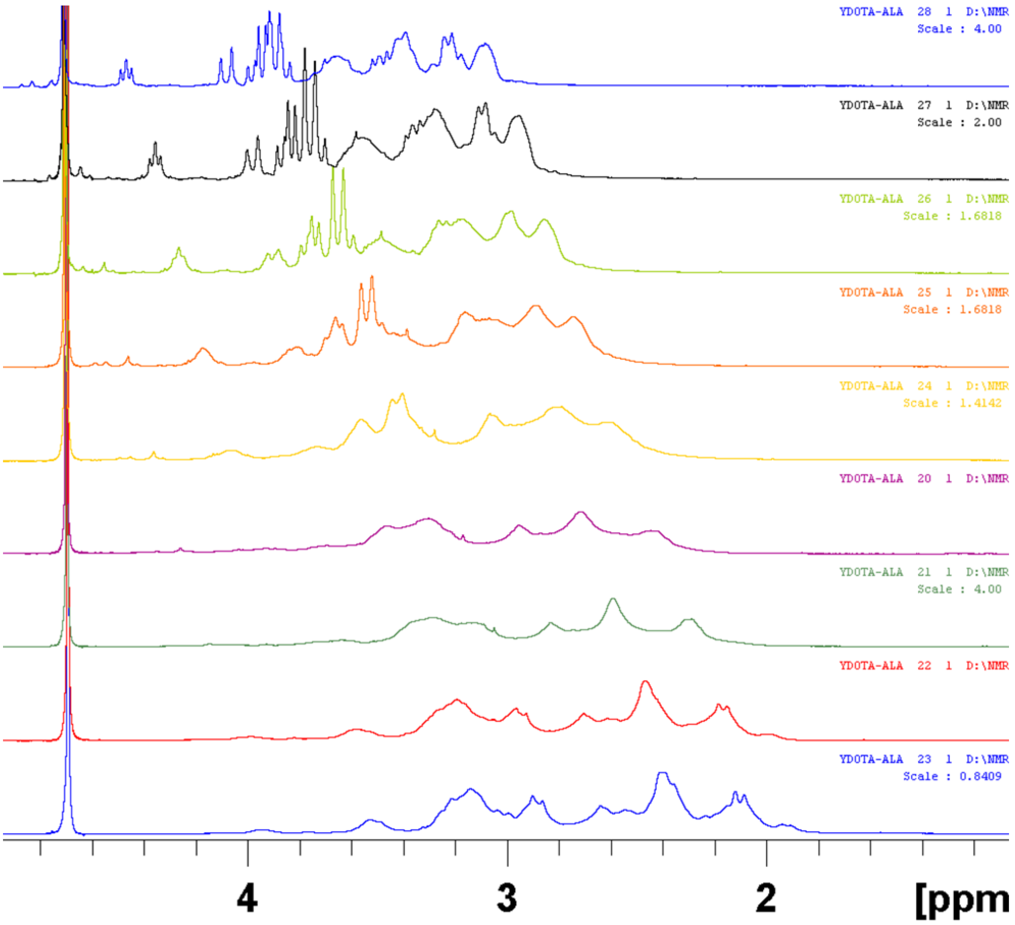

Figure S8. Temperature dependence of the ^{1}H NMR spectra of Y(DO3A-ACE)$^{-}$ in D_2O at 0, 7, 17, 27, 37, 47, 57, 67 and 80 ºC, from the bottom. (400 MHz, 20 mM, pD = 9.80).

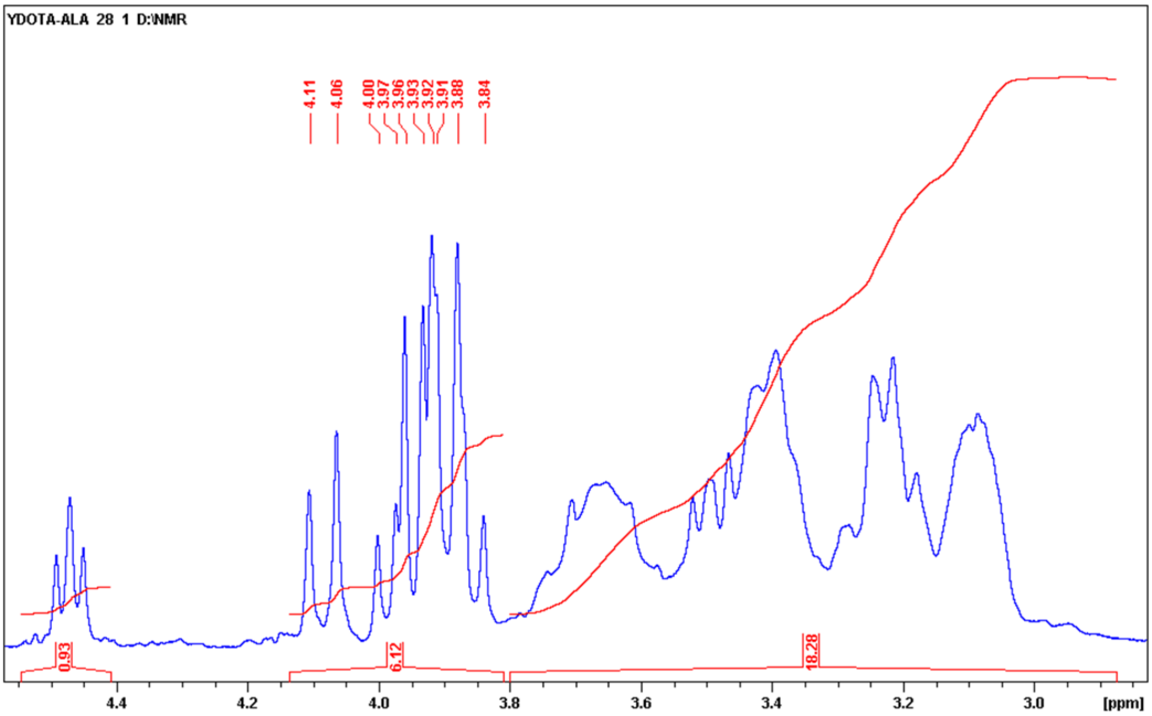

Figure S9. ^{1}H NMR spectrum and its integral for Y(DO3A-ACE)$^-$ in D_2O at 80 ºC (400 MHz, 20 mM, pD = 9.80).

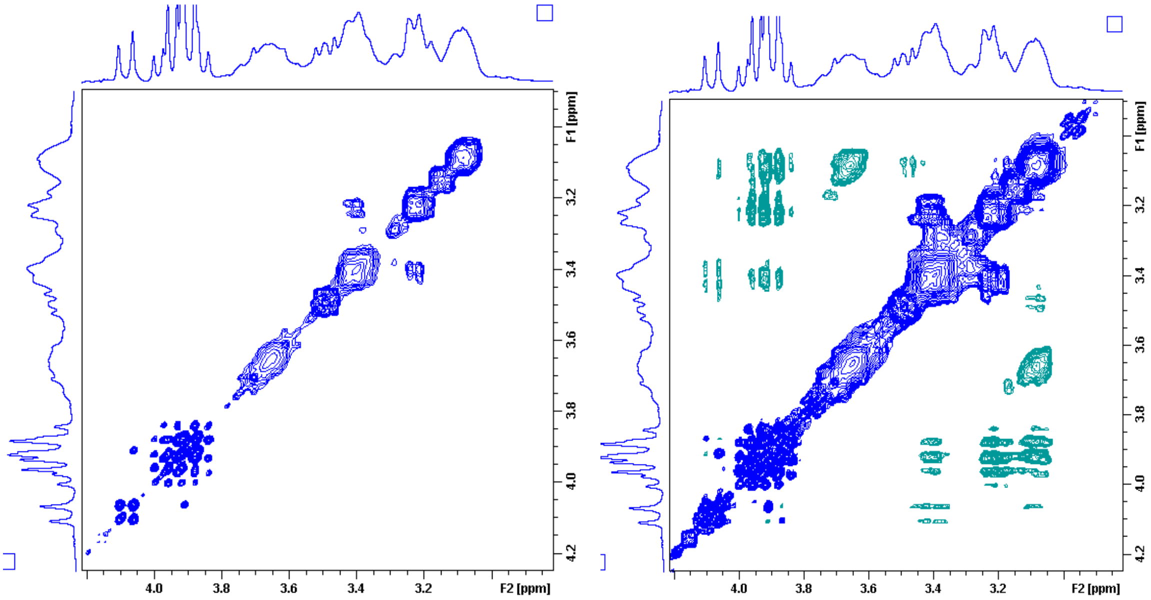

Figure S10. ^{1}H 2D NOESY spectra of the Y(DO3A-ACE)$^-$ complex at 80 ºC. At left only the strong peaks are shown, while at the right side the peaks are enlarged. Blue cross peaks are in same phase as the diagonal while the green ones are in opposite phase. Mixing time is 1 sec.

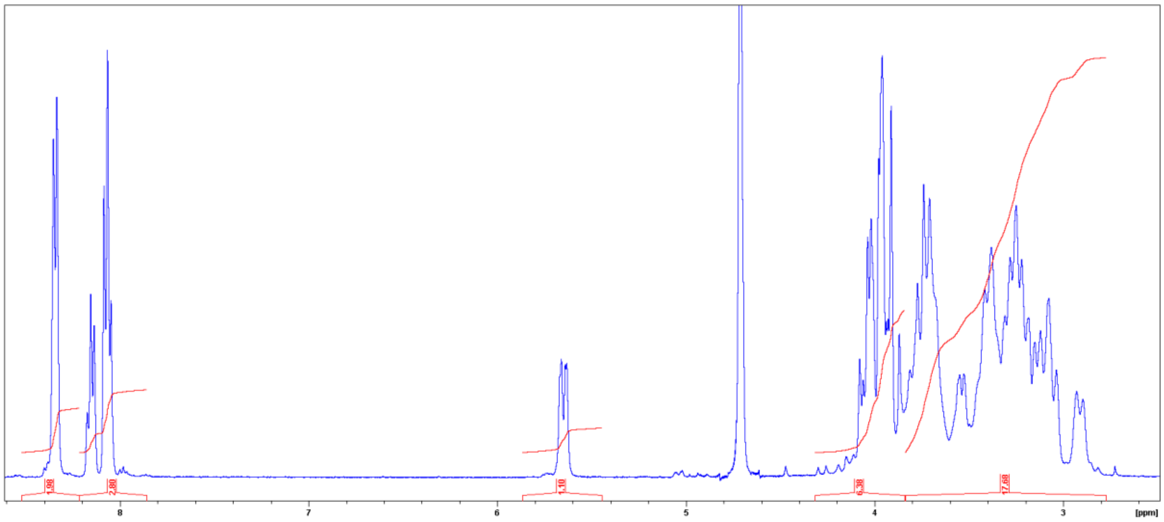

Figure S11. ^{1}H NMR spectrum and its integral for Y(DO3A-BACE)$^{-}$ in D_2O at 80 °C (400 MHz, 20 mM, pD = 9.80).

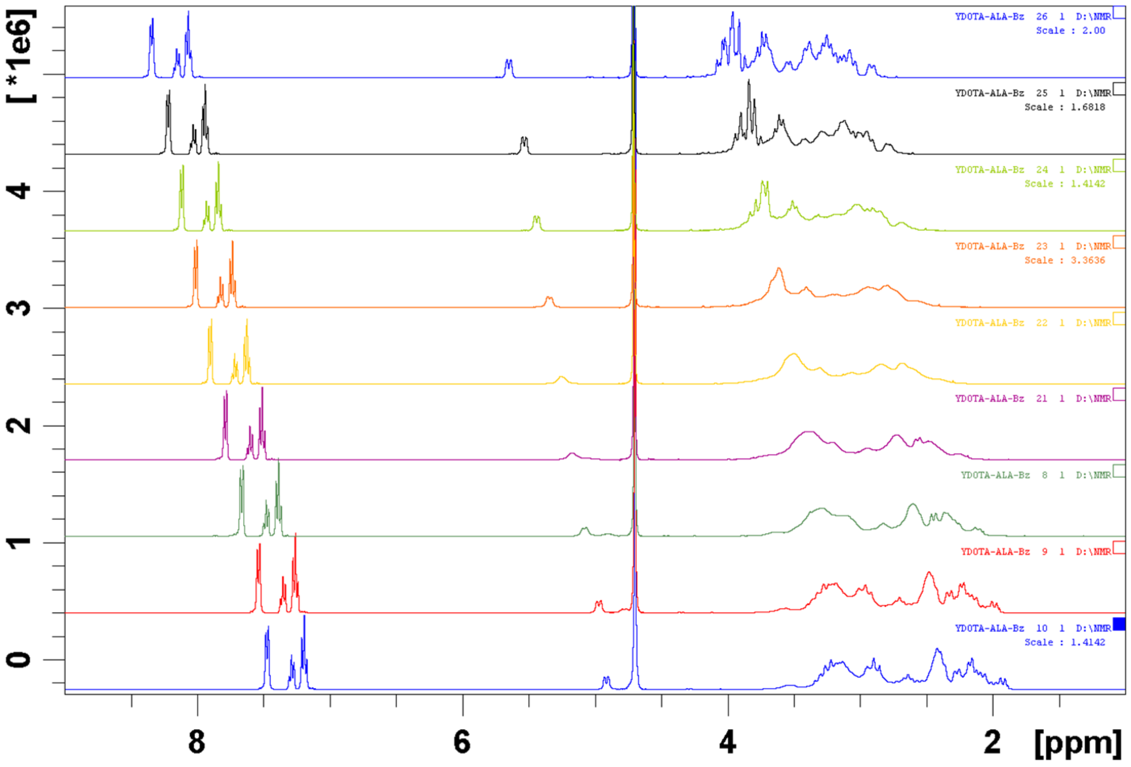

Figure S12. Temperature dependence of the ^{1}H NMR spectra of Y(DO3A-BACE)$^{-}$ in D_2O at 2, 7, 17, 27, 37, 47, 57, 67 and 80 °C, from the bottom. (400 MHz, 20 mM, pD = 9.80).

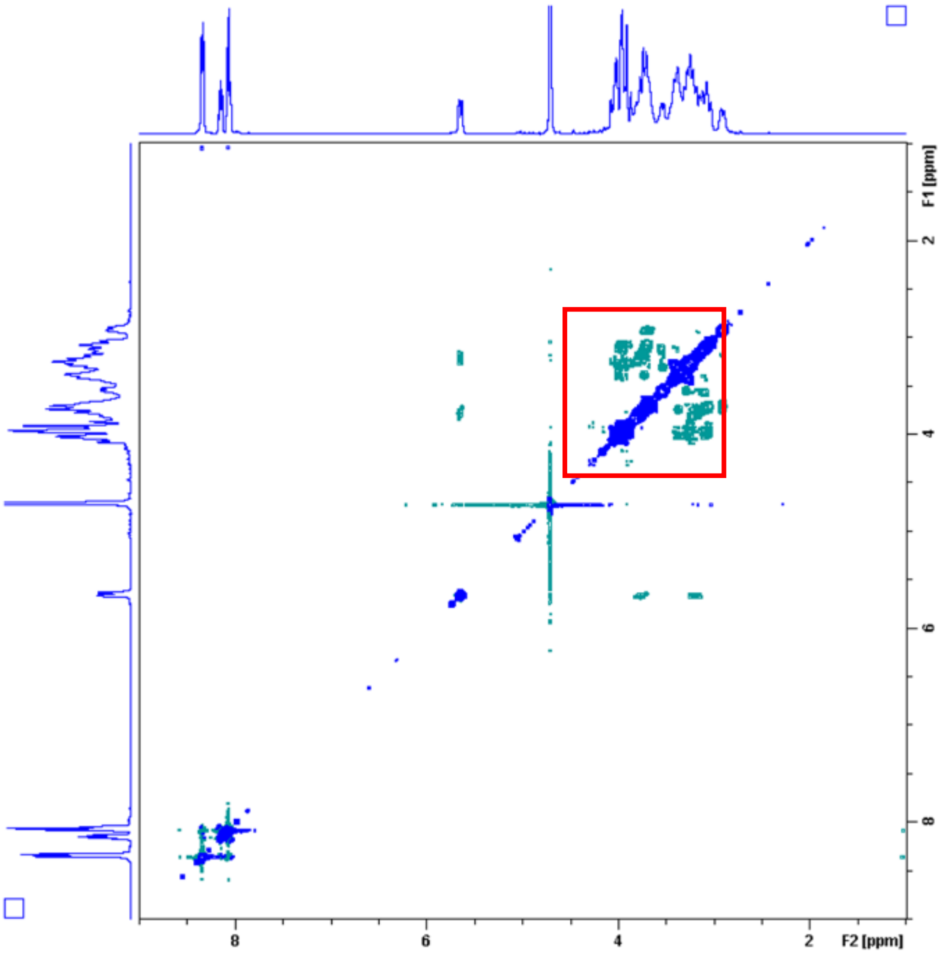

Figure S13. ^{1}H 2D NOESY spectrum of the Y(DO3A-BACE)$^{-}$ complex at 80 ºC. The red box shows the area enlarged in Figure 9. Blue cross peaks are in same phase as the diagonal while the green ones are in opposite phase.
